# Unanticipated benefits and potential ecological costs associated with pyramiding leafhopper resistance loci in rice

**DOI:** 10.1016/j.cropro.2018.09.013

**Published:** 2019-01

**Authors:** Finbarr G. Horgan, Maria-Liberty P. Almazan, Quynh Vu, Angelee Fame Ramal, Carmencita C. Bernal, Hideshi Yasui, Daisuke Fujita

**Affiliations:** a University of Technology Sydney, 15 Broadway, Ultimo, Sydney, NSW 2007, Australia; b Tropical Ecosystems Research Network, 30C Nirondha, Temple Road, Piliyandala, Sri Lanka; c International Rice Research Institute, DAPO Box 7777, Metro Manila, Philippines; d Cuulong Delta Rice Research Institute, Tan Thanh, Thoi Lai District, Can Tho, Viet Nam; e Helmholtz Centre for Environmental Research, Theodor-Leiser-Strasse, 06210, Halle, Germany; f School of Environmental Science and Management, University of the Philippines, Los Baños, 4030 Laguna, Philippines; g Plant Breeding Laboratory, Graduate School, Kyushu University, Fukuoka 812-8581, Japan; h Saga University, Faculty of Agriculture, 1 Honjo-machi, Saga, 840-8502, Japan

**Keywords:** Anti-herbivore resistance loci, Ecological costs, *Nephotettix virescens*, *Nilaparvata lugens*, Nitrogenous fertilizer, Planthoppers

## Abstract

We tested the hypotheses that increasing the number of anti-herbivore resistance loci in crop plants will increase resistance strength, increase the spectrum of resistance (the number of species affected), and increase resistance stability. We further examined the potential ecological costs of pyramiding resistance under benign environments. In our experiments, we used 14 near-isogenic rice lines with zero (T65: recurrent parent), one, two or three resistance loci introgressed through marker-assisted selection. Lines with two or more loci that were originally bred for resistance to the green rice leafhopper, *Nephotettix cincticeps*, significantly reduced egg-laying by the green leafhopper, *N. virescens*. Declines in egg-number and in nymph weight were correlated with the numbers of resistance loci in the rice lines. To test the spectrum of resistance, we challenged the lines with a range of phloem feeders including the zig-zag leafhopper, *Recilia dorsalis*, brown planthopper, *Nilaparvata lugens*, and whitebacked planthopper, *Sogatella furcifera*. There was an increase in the number of tested species showing significant declines in egg-laying and nymph survival on lines with increasing numbers of loci. In a screen house trial that varied rates of nitrogenous fertilizer, a line with three loci had stable resistance against the green leafhopper and the grain yields of infested plants were maintained or increased (overcompensation). Under benign conditions, plant growth and grain yields declined with increasing numbers of resistance loci. However, under field conditions with natural exposure to herbivores, there were no significant differences in final yields. Our results clearly indicate the benefits, including unanticipated benefits such as providing resistance against multiple herbivore species, of pyramiding anti-herbivore resistance genes/loci in crop plants. We discuss our results as part of a review of existing research on pyramided resistance against leafhoppers and planthoppers in rice. We suggest that potential ecological costs may be overcome by the careful selection of gene combinations for pyramiding, avoidance of high (potentially redundant) loci numbers, and introgression of loci into robust plant types such as hybrid rice varieties.

## Highlights

• Four hypotheses about the effects of pyramiding resistance genes in rice are tested.
• Pyramiding genes increased the strength of resistance against green leafhopper.
• Pyramiding genes increased resistance against a greater range of phloem feeders.
• Rice lines with three genes were most stable against leafhoppers under high nitrogen.
• Pyramiding resistance genes caused yield declines in benign environments.

## 1 Introduction

Recent advances in marker-assisted selection for food and fibre crops have streamlined breeding programs (Bourke et al., 2018; Garrido-Cardenas et al., 2018) and given new insights into the ecological and evolutionary relations between plants and components of their environment (O'Rourke et al., 2014; Odjo et al., 2017). One area where knowledge has rapidly accumulated is in the understanding of plant–herbivore interactions, particularly for monophagous and oligophagous phloem-feeding (Zhang et al., 2017) and gall-inducing (Sinha et al., 2017) insects that exhibit highly specific molecular challenge-and-defence interactions with their hosts. Where gaps in knowledge still exist, paradigms concerning anti-herbivore resistance are often borrowed from advances in disease resistance or from recent developments in transgenic research (Gould, 2003; Zhao et al., 2005; Horgan, 2018) without adequate empirical research using insect herbivores on conventionally bred varieties. Some paradigms, for example the maintenance of refuge areas to delay virulence adaptation, continue to govern current management practices for resistant crops without consideration of the inherent differences between transgenic resistance and conventional resistance based on ‘native’ genes (i.e., derived from the crop species or its wild relatives) (Horgan, 2018).

A range of phloem-feeding insects, including leafhoppers (e.g., the green rice leafhopper [GRH], *Nephottetix cincticeps*, and green leafhopper [GLH], *N. virescens*) and planthoppers (e.g., the brown planthopper [BPH], *Nilaparvata lugens*, and whitebacked planthopper [WBPH], *Sogatella furcifera*), currently challenge rice production in Asia. These species can cause severe yield reductions to intensified rice agroecosystems, particularly where farmers are encouraged to use high rates of nitrogenous fertilizer and prophylactic insecticide applications (Bottrell and Schoenly, 2012; Crisol et al., 2013). Host plant resistance has been the principal focus of public research into the management of rice leafhoppers and planthoppers for the last several decades and has gained new impetus from recent advances in molecular breeding (Fujita et al., 2013; Hu et al., 2016a). Because the success of host plant resistance is limited by herbivore adaptation to resistant varieties (often referred to as ‘virulence adaptation’), there has been a clear focus on selecting recognized ‘broad spectrum’ genes/loci, or on pyramiding a number of genes/loci into a single genotype to prolong resistance durability (Myint et al., 2012; Wang et al., 2017; Horgan et al., 2018a). A number of research groups have now developed multiple near-isogenic rice lines with a range of different resistance genes either singly introgressed (monogenic) or pyramided (polygenic) into high-yielding varieties, each with >85% recovery of the recurrent parent genes (Fujita et al., 2010; Hu et al., 2016b; Jena et al., 2017). Polygenic resistance to herbivores is predicted to be stronger (Fujita et al., 2010; Hu et al., 2013; Asano et al., 2015), have a broader spectrum (Hu et al., 2016b; Fan et al., 2017), have greater stability (Ali et al., 2006), and be more durable (Myint et al., 2012; Wang et al., 2017; Horgan et al., 2018a) than monogenic resistance. Ecological theory suggests that strong resistance is also likely to have associated costs as plants must balance growth, reproduction and defence (including maintenance) to achieve optimal life-history strategies in their respective niches (Herms and Mattson, 1992; Horgan et al., 2009). Research on traditional rice varieties with polygenic herbivore resistance supports many of these ideas (Hirae et al., 2007; Horgan et al., 2015, 2017). However, despite the tremendous investments into pyramiding herbivore resistance genes/loci, surprisingly few researchers have tested these hypotheses using available near-isogenic materials.

In the present study, we use a series of near-isogenic monolocus and pyramided (henceforth PYLs) lines to examine hypotheses concerning the number of herbivore resistance genes and the nature of resulting resistance. We developed clear tests to assess hypotheses concerning the strength, spectrum, stability and costs of resistance. We predicted that lines with higher numbers of genes/loci would have stronger resistance to phloem-feeders, irrespective of the identity of the genes. We predicted that lines with higher numbers of genes/loci would also affect a greater spectrum of phloem feeders due to the increasing probability of introducing non-specific resistance genes to lines through greater numbers of pyramided loci. Broad spectrum resistance is often evaluated in terms of the number of populations of a single species affected by the resistance. In our study, we examine the spectrum of resistance in terms of the number of different herbivore species affected by lines with increasing numbers of genes. Resistance stability can be measured as the consistency of resistance during different stages of plant growth or under different environments. We predicted that lines with increasing numbers of loci would maintain resistance and yields when attacked by leafhoppers under a gradient of fertilizer applications. Finally, we examined the potential costs of resistance for the rice plants. We predicted that under benign conditions (i.e., not challenged by herbivores), lines with higher numbers of loci would express trade-offs in plant growth and development or in reproductive output (yield). To our knowledge, this is the first study to test these hypotheses in a systematic manner using a collection of near-isogenic rice lines.

## 2 Materials and methods

### 2.1 Plant materials

A series of 14 near-isogenic lines (NILs) with Taichung 65 (T65), a *japonica* cultivar, as the genetic background were developed using marker-assisted selection and backcrossing. Four rice lines (IR24, DV85, IRGC104630, and IRGC105715) with recognized resistance to the green rice leafhopper (GRH), *N. cincticeps*, were used as donor parents. These were obtained from the Genetic Resources Center of the International Rice Research Institute (IRRI) in the Philippines. The following resistance loci have been identified and tagged from these donors: *GRH1* (from IR24, an *indica* variety: Yasui and Yoshimura, 1999), *GRH2* and *GRH4* (from DV85, an *indica* variety from Bangladesh: Yazawa et al., 1998), *GRH5* and *qGRH4* (from IRGC104630 [W1962], a wild accession of *Oryza rufipogon* from China: Fujita et al., 2006) and *GRH6* (from IRGC105715, a wild accession of *O. nivara* from Cambodia: Fujita et al., 2004).

Repeated backcrossing (three to six times) with T65 using marker-assisted selection produced *GRH1*-NIL (BC_3_), *GRH2*-NIL (BC_6_), *GRH4*-NIL (BC_6_), *GRH5*-NIL (BC_4_), *GRH6*-NIL (BC_3_), and *qGRH4*-NIL (BC_4_). Eight PYLs, *GRH2/GRH4*-PYL, *GRH2/GRH6*-PYL, *GRH4/GRH6*-PYL, *GRH5/qGRH4*-PYL*, GRH2/GRH5*-PYL, *GRH4/GRH5*-PYL, *GRH5/GRH6*-PYL, and *GRH2/GRH4/GRH6*-PYL were developed by crossing pairs of NILs with the corresponding GRH-resistance genes. The F_1_ plants carrying two or three GRH-resistance genes were self-pollinated and individuals with homozygous alleles at the resistance gene loci were selected through the F_2_ populations. Seed for the experiments was bulked-up in a screen house at IRRI during the dry season when temperatures were coolest.

The NILs and PYLs had previously been evaluated for resistance against GRH in Japan. Nymphal mortality on the lines was generally high (>74%) at two and eight weeks after sowing in seedling and leaf-blade bioassays, respectively (mortality on T65 ≤ 4%). Only *GRH4-NIL* did not exhibit antibiosis against nymphs in the tests (*GRH1*-NIL, *qGRH4*-NIL and *GRH5/qGRH4*-PYL were not evaluated: Fujita et al., 2010).

### 2.2 Herbivore species

We used two leafhopper species (GLH and the zig-zag leafhopper (ZLH), *Recilia dorsalis*) and two planthopper species (BPH and WBPH) in our experiments; however, GLH was the model species for most of our experiments because it was most affected by the resistance loci and is closely related to GRH. Colonies of all four species were initiated in 2008 using wild-caught individuals from Laguna Province in the Philippines (14°16′N, 121°21′E). Colonies were initiated with ca. 500 adults placed on TN1 (>30 DAS) in wire mesh cages of 120 × 60 × 60 cm (H × L × W) under greenhouse conditions (temperatures ranged from 25 to 37 °C, L12:D12 photoperiod). During the first two generations of rearing, the colonies were monitored for possible transmission of rice viruses. The leafhoppers did not transmit viruses.

### 2.3 Resistance strength

Aspects of resistance strength were assessed in greenhouse bioassays with GLH and BPH on potted plants. Germinated seeds of the 14 lines were each sown to size-0 (5 × 2.5 cm: H × R) pots under acetate cages (45 × 5 cm: H × R) and tended until 20-days after sowing (DAS), after which two gravid females were introduced to the cages. IR24 was included in the bioassays as an *indica* variety known to possess the *GRH1* gene. The females were allowed to oviposit for 5 days after which they were removed. Eggs were allowed to hatch and populations of nymphs developed during a further 10 days. Bioassays were replicated five times (15 lines × 2 species × 5 replicates = 150 pots). At the end of the bioassay, the nymphs were collected recording their developmental stages, were dried and weighed, and the plants examined under a microscope for any unhatched eggs.

Nymph survival in GLH was also examined in a greenhouse bioassay. The lines in size-0 zero pots under acetate cages (as above) were infested with ten newly-emerged nymphs (reared from TN1) at 20 DAS. Nymph survival was monitored daily and surviving nymphs collected at the end of 10 days. The bioassay was replicated five times (15 lines × 5 replicates = 75 pots). The developmental stages of the collected nymphs were recorded and the nymphs dried and weighed. Survival of BPH nymphs was not assessed because of a lack of apparent effects in preliminary experiments.

### 2.4 Resistance spectrum

The responses by two leafhoppers (GLH and ZLH) and two planthoppers (BPH and WBPH) to all 14 NILs were examined using two bioassays conducted in a controlled-temperature insectary (27 °C, D12:N12).

Nymph survival was monitored over 15 days in a test tube bioassay. Germinated seeds of the 14 lines were sterilized in 1% Chlorox for 20–30 min. After 2 days of soaking in water, the germinated seeds of each line were transferred to test tubes containing agar-Yoshida substrate (ratio: 10 g agar: 1 L Yoshida solution). Each test tube received a single rice seed. The test tubes were maintained in the insectary for 7 days. At 7 DAS, eight newly hatched nymphs were placed on the plants in each test tube with six replicates per line per insect species (14 lines × 4 species × 6 replicates = 336 test tubes). The conditions of the plants and nymph survival were recorded until 15 days after infestation (DAI). Where plants showed early signs of hopperburn (after 5–7 days), the bioassays were stopped and a final record of survival noted. Nymph biomass (dry weight) at 5 days (i.e., before hopperburn) on the 14 lines was estimated by collecting 5 day old nymphs fed on each line in separate test tubes under the same conditions (56 test tubes). The average biomass of nymphs on the fifth day in the main experiment (336 tubes) were then estimated by multiplying the number of survivors by the average individual dry weight on each line based on the 56 associated tubes.

Egg laying by each species was examined using potted plants (size-0 pots) each covered with a plastic acetate cage (as above). At 20 DAS, two gravid females were introduced to the cages. Infested plants were tended for 15 days, after which the number of emerged nymphs were counted and the plants were dissected under a microscope (magnification: 1.5–3) to count the unhatched eggs. The bioassay was replicated six times (14 lines × 4 species × 6 replicates = 336 plants).

### 2.5 Stability under gradients of nitrogen

We examined the effects of GLH infestations on the growth and yield of lines in three separate experiments. Each experiment was designed to investigate the effects of resistance genes in reducing plant damage and yield losses from GLH under gradients of fertilizer inputs. The first experiment was conducted in a greenhouse (using 12 lines) to examine aspects of host resistance under controlled conditions; however, because of the limitations of pot experiments in assessing fertilizer effects on yields (Crisol et al., 2013), the experiment was repeated in a screen house (using 8 lines) with a further experiment conducted in field plots (14 lines). The protocol and results from the greenhouse experiment are presented in the supplementary information. Only the screen house and field experiments are reported here.

#### 2.5.1 Screen house experiment

Seed of eight lines (T65, *GRH2*-NIL, *GRH4*-NIL, *GRH5*-NIL, *GRH2/GRH4*-PYL, *GRH2/GRH5*-PYL, *GRH2/GRH6*-PYL, and *GRH2/GRH4/GRH6*-PYL) were germinated in peat-filled trays. At 7 DAS, the germinated seedlings were transplanted to an outdoor screen house. The screen house was covered by 1 mm insect screening. The screen house contained a series of mud-filled concrete bays of 20 × 3 m (L × W) and 0.8 m deep and had paddy soil to a depth of ca 60 cm that reached the original topsoil (the bays did not have a concrete base). Five bays were each separated into three plots using dividing timbers. The plots were further surrounded by earthen levees to prevent flooding inside the plots. Plots received one of three fertilizer regimes (equivalent to 0, 60 and 150 Kg N ha-1) as two applications (basal and at 15 DAS) of ammonium sulphate.

Rice seedlings were randomly planted to each plot (two of each line, 16 seedlings per plot) with a distance of 50 cm between seedlings. The seedlings were individually enclosed in insect cages. The cages were constructed using cylindrical, wire frames of 150 × 20 cm (H × R) covered in insect-proof mesh. The mesh, draped over the frames, was glued at the base to a cylindrical plastic sheet 20 cm high. Plastic sheets were driven into the paddy soil to secure the cages. At 20 DAS (early tillering), 4 gravid female GLH were introduced to half of the cages in each plot, the remaining cages maintained non-infested control plants (8 lines × 2 infestation levels × 3 nitrogen regimes (plots) × 5 screen house bays = 240 cages). Plants were tended until they began to turn yellow (prior to hopperburn) or were ready for harvest (>85% of grain matured) at which time they were destructively sampled.

At sampling, the GLH were collected from the plants using a vacuum sampler (Hausherr's Machine Works, USA). The number of tillers were counted (distinguishing productive and non-productive tillers) and the plants destructively harvested. Plant parts were separated as roots, shoots and panicles into paper bags. The plants and GLH were dried in a forced draught oven at 60 °C for >1 week and weighed. Grain was removed from the panicles and separated as filled and unfilled grain before counting and final weighing.

#### 2.5.2 Field exposures

Fourteen lines were grown under field conditions at the IRRI Experimental Field Station (Los Baños, Laguna). The site has deep clay soils with about 4% organic matter. The experiment was conducted in a series of 12 rice plots that received one of two nitrogen regimes: zero added nitrogen (0 Kg N ha^−1^) and 60 Kg N ha^−1^. Treatments were replicated in six fields (two plots/nitrogen treatments per field). Separate sub-irrigation channels were installed around each plot. These connected to the main field canals for irrigation and drainage, but prevented leakage of nutrients among adjacent fields or among plots within fields.

At the time of the experiment, the fields were planted with IR66. The near-isogenic lines were planted as an embedded experiment among the IR66 in plots with 2 × 6 rows of each line (plot size = 8 × 1.75 m including rice-free boundaries). The experiment followed guidelines for embedded field trials as reported by Horgan et al. (2018b). Seed was initially sown in seedling trays for germination. At 20 DAS, the seedlings were transplanted to the field plots at 1 seedling per hill. Hills were separated by 25 cm (spacing = 25 × 25 cm). Plots with added nitrogen had three ammonium sulphate top dressings, the first applied basally, with further applications at the mid-tillering stage and before flowering. Solophos, muriate of potash and zinc were applied basally with the ammonium sulphate. No pesticides were used in the fields at any time.

Plants were destructively harvested when grain was >85% mature. The harvested plants were divided into separate plant parts as described above (2.5.1) and were dried and weighted. The numbers of tillers and panicles and the proportions of filled and unfilled grain were also noted.

### 2.6 Data analyses

Results from greenhouse fitness tests were examined using univariate general linear models (GLM) with Dunnett's many-to-one post-hoc comparisons against T65. Development of BPH on 20 DAS plants was examined using multivariate GLM. Nymph biomass was log-transformed and proportions arcsine-transformed.

The spectrum of resistance was examined using univariate GLM with lines, herbivore species and their interaction as main factors. Because of differences in fecundity and nymph biomass between hopper species, the proportional reductions in these fitness parameters for hoppers on resistant lines compared to each species on T65 were calculated. Pair-wise comparisons across herbivore species and for proportional reductions were conducted using Tukey tests. Otherwise pair-wise comparisons of lines (days to wilt and survival) were compared using 2-tailed Dunnett's many-to-one comparisons. Egg numbers were log-transformed and survival rates arsine-transformed before analyses. Univariate GLMs were conducted for each species and fitness parameter.

For the screen house experiment, GLH pressure was expressed as the biomass of leafhoppers per unit weight of plant. Data from the experiments was analysed using univariate GLMs for a split-split plot design with nitrogen regime (main plot), line, infestation/control and their interactions as main factors. Proportional changes in plant growth and productivity under control and infested conditions in the screen house were estimated as *(Infested-Control)/Control* and analysed as a split-plot experiment. Herbivore pressure was not assessed in the field plot experiment. The field data were analysed as a split plot experiment (factors = nitrogen [main plot], lines and their interaction).

We assessed the relations between resistance and plant development using Pearson's correlations. Where data was not normal or homogenized we used Spearman's rank correlations. Data residuals for parametric tests were plotted after each analysis and verified as normal and homogeneous. Analyses were conducted using SPSS v.22 (IBM SPSS, Armonk, NK, USA).

## 3 Results

### 3.1 Resistance strength

GLH nymphs had lower survival and nymph biomass on *GRH2/GRH4*-PYL and *GRH2/GRH4/GRH6*-PYL compared to T65 (P < 0.05, Dunnett's test, Table 1). Survival of GLH nymphs on 20 DAS *GRH5/qGRH6*-PYL was also lower (Table 1).

**Table 1:** Results of bioassays with *Nephottetix virescens* on 14 near-isogenic rice lines with zero, one, two or three resistance loci. See Table S1 for a similar experiment with *Nilaparvata lugens*.

Rice line^a^	Oviposition bioassay^b^			Nymph survival bioassay^b^	
	Total eggs laid^c^	Prop. Unhatched eggs^d^	Nymph weight (mg)	Prop. Survival^d^	Nymph weight (mg)^c^
T65	104.40 ± 16.96	0.08 ± 0.06	3.03 ± 0.38	0.95 ± 0.03	0.26 ± 0.04
IR24 (*GRH1*)	87.75 ± 15.48	0.04 ± 0.03	3.03 ± 0.79	0.95 ± 0.03	0.26 ± 0.03
*GRH1-*NIL	120.60 ± 21.23	0.06 ± 0.04	3.76 ± 0.44	0.95 ± 0.03	0.27 ± 0.02
*GRH2-*NIL	80.40 ± 14.35	0.02 ± 0.02	2.80 ± 0.22	0.90 ± 0.07	0.30 ± 0.07
*GRH4-*NIL	92.60 ± 27.34	0.02 ± 0.01	3.42 ± 0.63	0.93 ± 0.05	0.35 ± 0.08
*GRH5-*NIL	153.00 ± 25.01	0.02 ± 0.01	4.36 ± 0.33	0.93 ± 0.03	0.23 ± 0.03
*GRH6-*NIL	58.40 ± 9.56	0.06 ± 0.03	2.21 ± 0.29	0.93 ± 0.05	0.33 ± 0.06
*qGRH4-*NIL	42.25 ± 5.24**	0.02 ± 0.01	1.38 ± 0.25*	0.68 ± 0.15	0.21 ± 0.03
*GRH2/GRH4*-PYL	30.40 ± 5.94***	0.00 ± 0.00	1.04 ± 0.18**	0.28 ± 0.09***	0.08 ± 0.03*
*GRH2/GRH5*-PYL	41.50 ± 8.95**	0.05 ± 0.01	1.37 ± 0.37*	0.90 ± 0.06	0.32 ± 0.08
*GRH2/GRH6*-PYL	45.60 ± 5.27*	0.14 ± 0.05	1.46 ± 0.20*	0.75 ± 0.19	0.29 ± 0.02
*GRH4/GRH5*-PYL	93.00 ± 18.25	0.04 ± 0.02	3.83 ± 0.39	0.80 ± 0.15	0.29 ± 0.06
*GRH4/GRH6*-PYL	65.25 ± 3.77	0.00 ± 0.00	2.61 ± 0.12	0.88 ± 0.00	0.27 ± 0.01
*GRH5/qGRH4*-PYL	78.20 ± 5.75	0.04 ± 0.03	2.99 ± 0.26	0.48 ± 0.18**	0.15 ± 0.06
*GRH2/GRH4/GRH6*-PYL	28.00 ± 3.44***	0.19 ± 0.14	1.01 ± 0.33***	0.18 ± 0.09***	0.05 ± 0.02**
F_14,60_-value	6.204***	1.452ns	8.026***	7.305***	3.948***

a IR24 containing the *GRH1* gene was included as an *indica* check variety and is not isogenic with T65.

b Numbers are means ± SEM (N = 5);*** = P ≤ 0.001,** = P ≤ 0.01,* = P ≤ 0.05, ns = P ≥ 0.05; asterisks adjacent to SEMs are based on Duncan's many-to-one comparisons.

c Data log (x+1) transformed.

d Data arcsine transformed.

Compared to T65, egg-laying by GLH was lower on *qGRH4*-NIL, *GRH2/GRH4*-PYL, *GRH2/GRH5*-PYL, *GRH2/GRH6*-PYL and on *GRH2/GRH4/GRH6*-PYL, (Table 1). Results from the greenhouse bioassays with GLH indicated that some of the egg mortality in *GRH2/GRH6*-PYL and *GRH2/GRH4/GRH6*-PYL may be due to plant ovicidal effects that resulted in higher (though not significantly: F_14,60_ = 1.452, P > 0.05) proportions of unhatched eggs (Table 1).

**Fig. 1:**
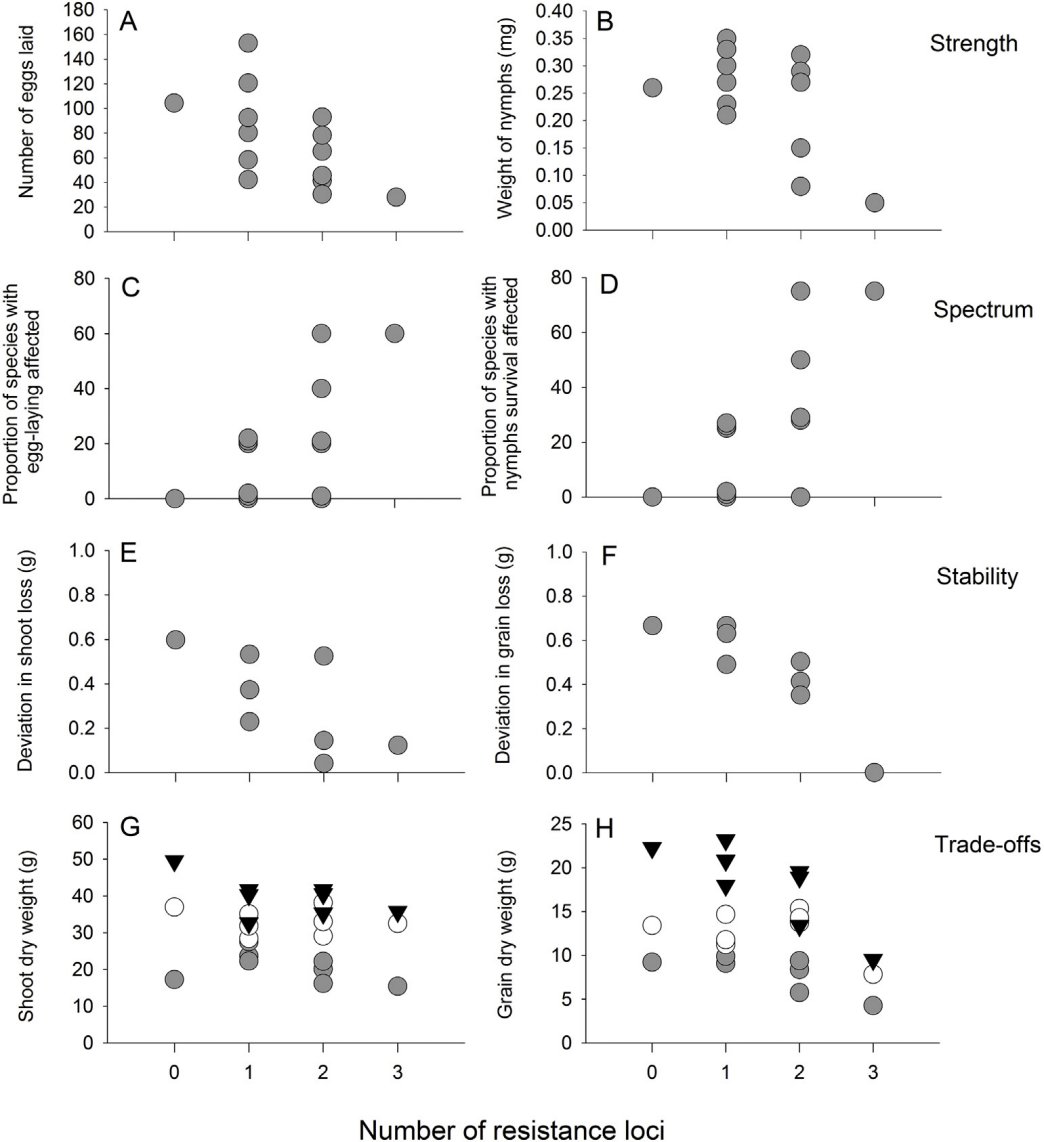
Correlations between the number of resistance loci in rice lines and A) the number of eggs laid by GLH, B) the weight of GLH nymphs after 10 days on rice seedlings, C) the proportion of hopper species with significant reductions in egg laying (from a total of 4 species), D) the proportion of hopper species with<50% nymph survival on rice seedlings (from a total of 5 species), E) the mean deviation from zero loss (= no loss, based on control) of shoot biomass in plants infested with GLH across a nitrogen gradient ([[shoot loss at 150 Kg N ha^−1^ – shoot loss at 60 Kg N ha^−1^] + etc.]/3); F) the mean deviation from zero in grain loss across a nitrogen gradient ([[grain loss at 150 Kg N ha^−1^ – grain loss at 60 Kg N ha^−1^] + etc.]/3); G) The weight of shoots under benign conditions; and H) the yield of plants under benign conditions. Shaded symbols in G and H = 0 Kg N ha^−1^, open symbols = 60 Kg N ha^−1^, black symbols = 150 Kg N ha^−1^.

The average number of eggs laid by GLH on the lines was negatively correlated with the number of resistance loci (C_14_ = −0.586; P = 0.028: Fig. 1 A). Nymph weights were also negatively correlated with the number of resistance loci (C_14_ = −0.695, P = 0.006: Fig. 1 B), but nymph survival was not correlated with the number of loci (C_14_ = −0.511, P = 0.062).

In the greenhouse bioassay, a decrease in egg-laying by BPH resulted in lower weights of nymphs on *GRH2/GRH6*-PYL and *GRH2/GRH4/GRH6*-PYL (F_14,75_ = 5.373, P < 0.001). The host lines had no effect on BPH nymph development (Wilk's F_42,217_ = 1.075, P > 0.05; between subject effects: 1.321 > F_14,75_ > 1.181, P > 0.05: Table S1). Egg-laying in BPH was not correlated with the number of loci (C_14_ = −0.261, P = 0.367).

### 3.2 Resistance spectrum

**Fig. 2:**
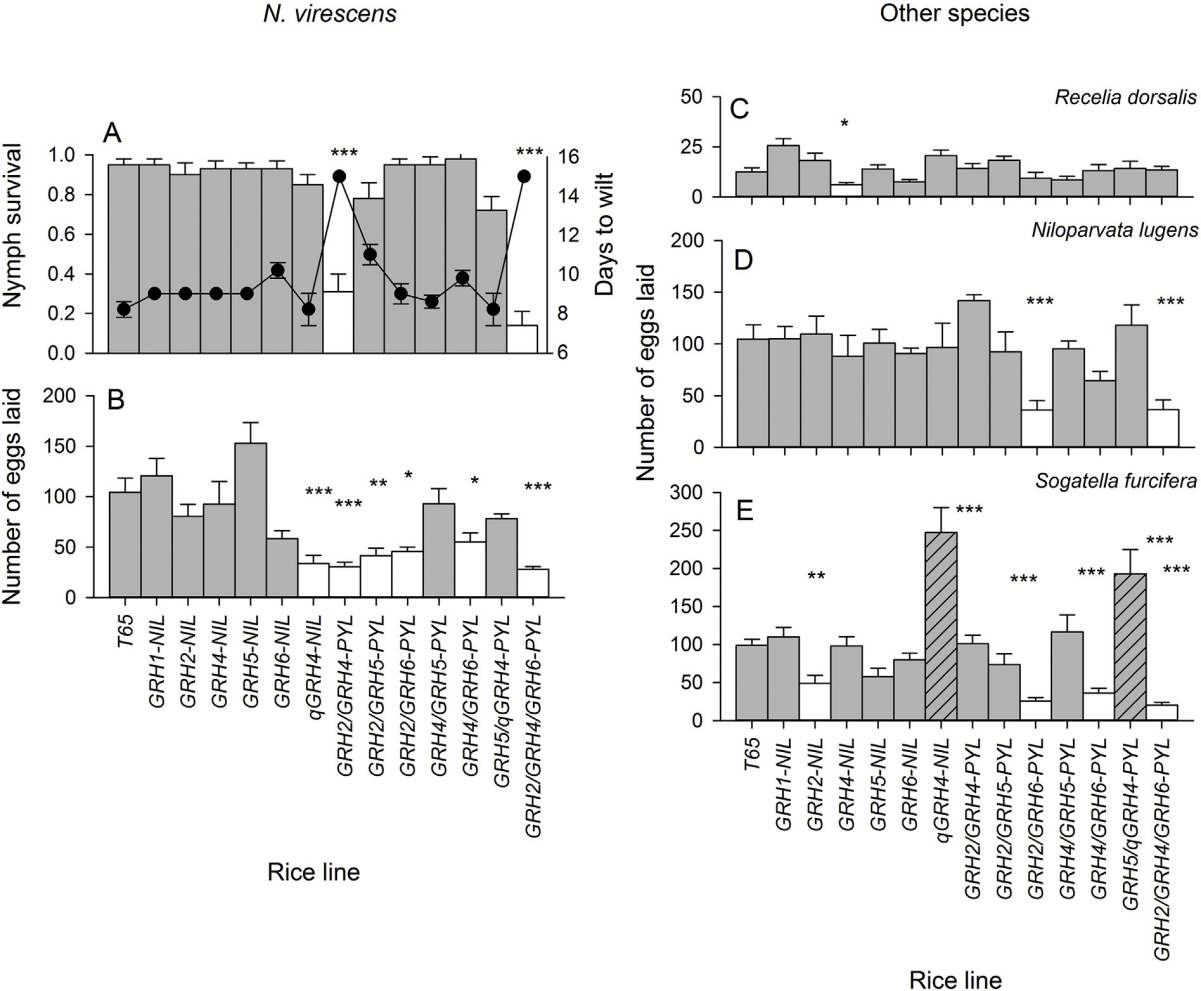
A) Proportion of nymphs surviving (bars) and number of days for 7 DAS plants to wilt (solid points) when infested with GLH nymphs and numbers of eggs laid by gravid female GLH (B), ZLH (C), BPH (D) and WBPH (E) on plants infested at 20 DAS. Standard errors are indicated (N = 6). Asterisks indicate significant reductions in herbivore fitness compared to T65 (Dunnett's tests: *** = P ≤ 0.001, ** = P ≤ 0.01, * = P ≤ 0.05). Resistant genotypes are indicated by white bars; cross-hatching in E indicates significant increases in egg laying (see Table S2A and S2B for further details).

Resistance loci variously affected nymph feeding across the range of phloem feeders (Days to wilt: F_13,280_ = 17.453, P < 0.001 [*GRH2/GRH4*-PYL, *GRH2/GRH5*-PYL and *GRH2/GRH4/GRH6*-PLY < T65]; nymph survival: F_13,280_ = 4.090, P < 0.001 [*GRH2/GRH4-*PYL, and *GRH2/GRH4/GRH6*-PYL < T65]; nymph biomass: F_12,260_ = 3.505, P < 0.001 (*GRH2/GRH5*-PYL < T65]: Tables S2A and S2B). However, the resistant lines were only effective against nymph feeding in GLH (days to wilt: F_3,280_ = 416.332, P < 0.001; nymph survival: F_3,280_ = 39.283, P < 0.001; nymph biomass: F_3,260_ = 3.505, P < 0.001: Fig. 2 A; Tables S2A and S2B). This produced significant line × herbivore interactions (days to wilt: F_39,280_ = 16.669, P < 0.001; nymph survival: F_39,280_ = 3.723, P < 0.001; nymph biomass: F_36,260_ = 3.187, P < 0.001) (see Fig. 2; Tables S2A and S2B for full details of results).

Rice lines variously affected egg-laying by each herbivore species (F_12,260_ = 12.697, P < 0.001) with *GRH4*-NIL, *GRH6*-NIL, *GRH4/GRH6*-PYL, *GRH2/GRH4*-PYL and *GRH2/GRH4/GRH6*-PYL forming the most resistant group [Tukey = P < 0.05]). Among the herbivore species, GLH (Fig. 2 B) was more severely affected by resistant lines than BPH and WBPH, with ZLH largely unaffected (F_3,260_ = 17.649, P < 0.001: Fig. 2C–E). There was a significant interaction term (F_36,260_ = 6.230, P < 0.001) due to the varying effects of resistance loci on different herbivores (see Table 2A for further details). Egg-laying by GLH, BPH and WBPH was lower on *GRH2/GRH6*-PYL and *GRH2/GRH4/GRH6*-PYL than on T65. Egg-laying by GLH and WBPH were also lower on *GRH4/GRH6*-PYL. GLH also laid fewer eggs on *qGRH4*-NIL, *GRH2/GRH4*-PYL and *GRH2/GRH5*-PYL that on T65 (Fig. 2B–E). WBPH laid more eggs on *qGRH4*-NIL and *GRH5/qGRH4*-PYL than on T65 (Fig. 2 E).

**Table 2:** Summary results of studies that test the relation between the number of planthopper and leafhopper resistance loci introgressed into rice plants and the comparative strength, spectrum, stability, durability and costs of polygenic resistance.

Loci/genes^a^	Number of plants			Hypothesis	Evidence^b^	Source
	1 locus	2 loci	3 loci			
Brown planthopper						
*BPH14, BPH15*	2	1	–	Greater strength	Antibiosis - adult female survival, sex-ratios and the proportion of females that were winged were lower on PYL than on NILs; Antixenosis - lower settling on PYL compared to NILs	Li et al. (2011)
*BPH14, BPH15, BPH18*	3	2	1	Greater strength	Antibiosis - in honeydew excretion tests, 3 loci = 2 mg, 2 loci = 2.5–7.5 mg, 1 locus = 4–10 mg, recurrent parent = > 20mg/female; nymph mortality on seedlings, 3 loci = 80%, 2 loci = 78-45%, 1 locus = 55-38%, recurrent parent = 14%	Hu et al. (2013)
				Inherent trade-offs	Inconclusive (field trials without record of herbivore pressure)	
*BPH25, BPH26*	2	1	–	Greater strength	Antibiosis - lower weights and slower development of nymphs on PYL compared to resistant NILs	Myint et al. (2009)
				Broader spectrum	Antibiosis (nymph and adult survival and weight gain, swollen female abdomens) of PYL observed against 4 colonies, but antibiosis of NILs only against 2 colonies	
*BPH25, BPH26*	2	1	–	Greater strength	Antibiosis - adult mortality on cut leaves, 2 loci = 80%, 1 locus = 10–15%, recurrent parent = > 10%, adult females with swollen abdomens, 2 loci = 0%, 1 locus = 60–80%, recurrent parent = 80%	Myint et al. (2012)
*BPH6, BPH12*	2	1	–	Greater strength	Antibiosis - population growth was 20% lower on PYL than on best NIL; Antixenosis - settling by nymphs after 120 h was similar on PYL and best NIL but lower than the recurrent parent (40%). PYLs and NILs were not directly compared in binary choice tests	Qui et al. (2012)
*BPH3*, *BPH27(t)*	2	3	–	Greater strength	Inconclusive because resistance and tolerance are confounded in seedling tests	Liu et al. (2016)
				Inherent trade-offs	Field plot studies indicated that PYLs and NILs had lower yields than the recurrent parent, but yields were comparatively higher that the parent when infested with BPH	
*BPH2, BPH4, BPH9, BPH10, BPH17, BPH18, BPH20, BPH21, BPH26, BPH32*	9	11	5	Greater strength	Antibiosis - larger proportions of PYLs had < 500 mm^2^ of phloem-honeydew excretion (3 loci = 100%, 2 loci = 100%, 1 locus = 67%; however, the authors did not compare results directly	Jena et al. (2017)
				Broader spectrum	Honeydew excretion by 1 of 4 BPH populations was equivalent to the recurrent parent on 3 NILs; however, honeydew production by all 4 populations was reduced on all PYLs	
				Inherent trade-offs	Inconclusive (study does not report herbivore pressure or grain yields)	
*BPH6, BPH9*	2	1	–	Greater strength	Antibiosis - weight gain by nymphs was lower on PYL (0.3 mg), than on either NIL (0.5 mg; recurrent parent = 1.2 mg). Similar results with honeydew excretion bioassays; Antixenosis - about half the number of nymphs settled on PYL compared to NILs after 120 h	Wang et al. (2017)
				Inherent trade-offs	Inconclusive (field trials without record of herbivore pressure)	
*BPH3, BPH14, BPH15, BPH18, BPH20, BPH21*	6	1	–	Greater strength	Antibiosis - honeydew excretion on PYL was <60% of than on best NILs; higher mortality of nymphs on PYL seedlings (ca 80%) than on best NILs (35–40%).	Jiang et al. (2018)
				Inherent trade-offs	Inconclusive (field trials without record of herbivore pressure)	
*BPH14, BPH15*	2	1	–	Greater strength	Antibiosis - honeydew excretion and population growth were lowest on PYL; Antixenosis - female settling rates on PYL were about 50% of those on NILs	Han et al. (2018)
Green leafhopper						
*GRH1, GRH2*, *GRH4, GRH5, qGRH4, GRH6*	6	3	–	Greater strength	Antibiosis - nymph mortality on seedlings was 15–25% higher on PYLs compared to the strongest NILs	Fujita et al. (2010)
*GRH2, GRH4*	2	1	–	Greater strength	Antibiosis - nymph and adult survival and biomass on PYL were <30% of that on NILs; Antixenosis - egg-laying on PYL was <50% of that on NILs; settling by nymphs on PYL was <20% of than on NILs	Vu et al. (2014)
				Greater stability	*GRH2/GRH4-PYL* maintained resistance throughout development. *GRH4-NIL* had moderate resistance at 60 days after sowing, but was otherwise susceptible	
				Inherent trade-offs	Inconclusive (experiment with potted plants)	
*GRH2, GRH4*				Greater durability	Selection on susceptible, monogenic NILs resulted in virulence against the PYL; although inconclusive as a test of the durability hypothesis, this does suggest that 2-loci PYLs are as vulnerable as monogenic NILs to virulence adaptation in this case	Horgan et al. (2018a)
*GRH1, GRH2*, *GRH4, GRH5, qGRH4, GRH6*	6	6	1	Greater strength	Antibiosis – GLH nymph survival negatively correlated with the number of resistance loci; Antixenosis – GLH egg-laying negatively correlated with the number of resistance loci	Present study
				Broader spectrum	3-loci PYL reduced egg laying in GLH, BPH, and WBPH, 2-loci PYLs reduced egg laying in GLH (4/6 PYLs), BPH (1/6 PYLs) and WBPH (2/6 NILs). I/6 NILs reduced egg laying in either GLH, ZLH or WBPH; the number of species affected was positively correlated with the number of resistance loci	
				Greater stability	GLH biomass densities remained low (<1.43 mg/g) on 3-loci PYL and one of three 2-loci PYLs (<0.073 mg/g) across a gradient of fertilizer applications. Biomass density varied between 1.35-7.92 and 4.83–10.14 mg/g on the remaining 2-loci NILs.	
				Inherent trade-offs	Yields were negatively correlated with the number of resistance loci under low and high nitrogen in the absence of herbivores under screen house conditions	

a Loci abbreviations are changed to uppercase from some of the original publications; however, loci numbers are maintained in accordance with the original publications, except *BPH25* and *BPH26* which were changed from *Bph20 (t)* and *Bph21 (t)* in some early publications to avoid confusion with *Bph20* and *Bph21* as in Jiang et al. (2018).

b Most authors have reported similar or higher yields and little change in agronomic performance of pyramided lines compared to recurrent parents; however, potential yield and/or other agronomic penalties cannot be tested in unprotected field plot experiments. Results from studies with potted plants are also inconclusive because of unpredictable effects of pot restrictions on growth and yield (Crisol et al., 2013). Publications and experiments that evaluate plants using seedling bulk tests and tests of plant growth response have not been included because such tests confound plant resistance and tolerance (i.e., Sharma et al., 2004, Hu et al., 2015, 2016b). Tests that only compared PYLs to recurrent parents are also excluded (Hu et al., 2012; Xu, 2013; Wang et al., 2016; Xiao et al., 2016; Fan et al., 2017).

The numbers of herbivore species with nymph mortality above 50% (including published data from seedling tests with *N. cincticeps*: Fujita et al 2010) and with significant reductions in egg-laying compared to phloem feeders on T65 were correlated with the number of loci in each line (Spearman's R_14_ = 0.531, P = 0.051 for both nymph mortality and egg-laying reduction: Fig. 1B and C).

### 3.3 Resistance and productivity under gradients of nitrogen

Results from the greenhouse experiment are presented in Tables S3A and S3B together with four extra rice lines that were not included in the controlled screen house experiments.

**Fig. 3:**
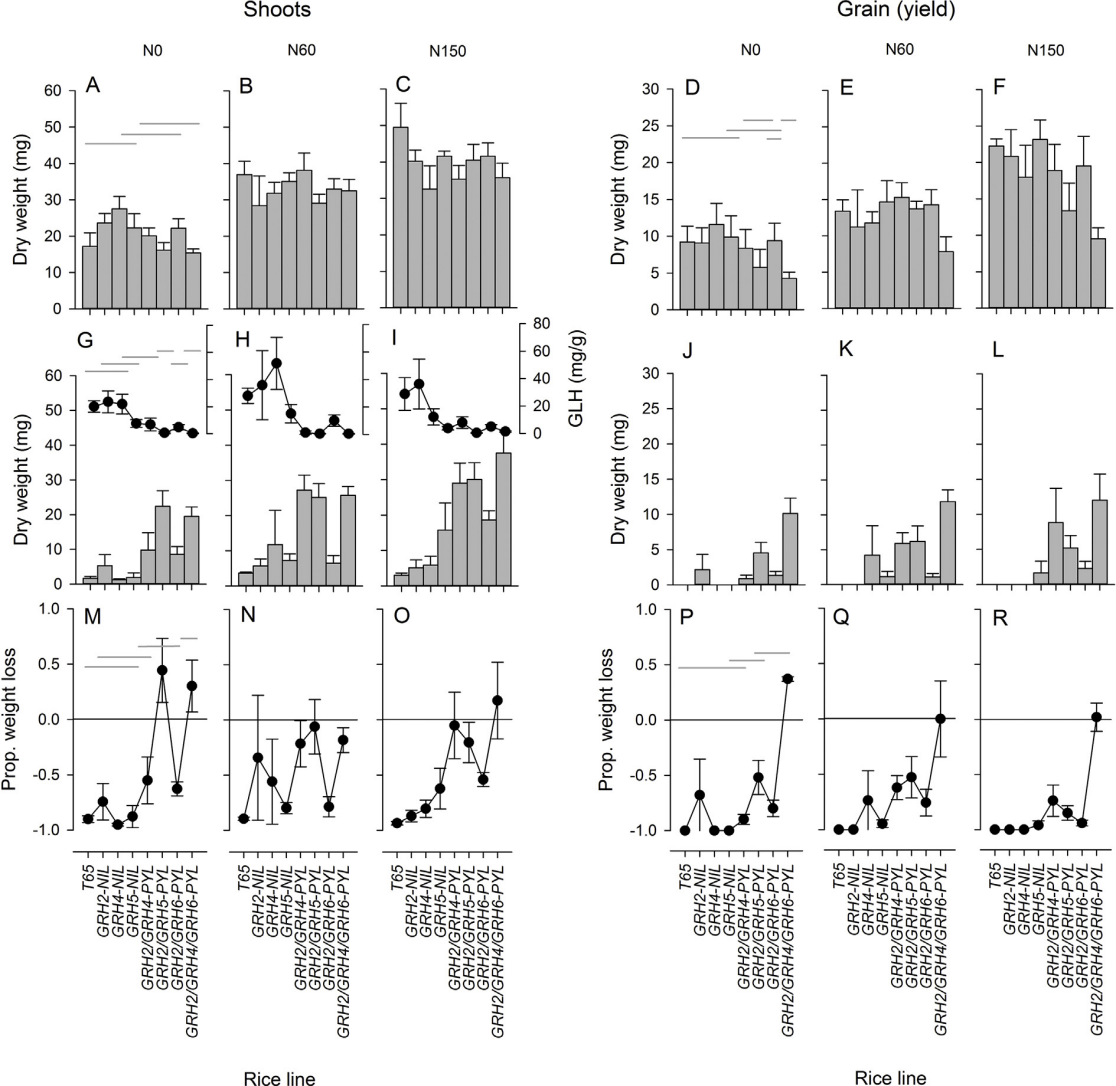
The main results from the screenhouse experiment. Graphs indicate the biomass of shoots (A-C, G-I) and grain (D-F, J-L) of eight lines grown under three nitrogen regimes - zero added nitrogen (A, D, G, J), 60 Kg N ha^−1^ added (B, E, H, K) and 150 Kg N ha^−1^ added (C, F, I, L) – that were either without herbivores (A–F) or infested with *Nephotettx virescens* (GLH) (G–L). The corresponding biomass densities of GLH on infested plants are indicated at the top portions of G-I. The proportional losses in shoot (M–O) and grain (P–R) biomass due to GLH are indicated. Points above the x = zero line in M,O,P and Q indicate overcompensation for GLH attack, points below the line are proportional losses. Homogenous shoot (A), grain (D), GLH biomass density (G), proportional biomass loss (M) and proportional yield loss (P) are indicated by gray lines. Standard errors are indicated (N = 5). For further details see Table S4. Greenhouse and field experiments are indicated in Tables S3 and S5, respectively.

Nitrogen increased growth and grain yields under screen house conditions (Fig. 3). Grain size (100 grain weight), and the proportions of productive tillers or filled grain were not affected by nitrogen (Table S4A). GLH infestations reduced plant growth (Fig. 3G–I) and yield (Fig. 3J–L) but had no effect on the proportions of tillers that were productive or the proportions of grain that were filled (Table S4A and S4B). GLH attained lower population densities on *GRH5*-NIL, *GRH2/GRH4*-PYL, *GRH2/GRH5*-PYL, *GRH2/GRH6*-PYL and *GRH2/GRH4/GRH6*-PYL compared to T65 (Fig. 3G–I). Rice line had little effect on plant production under control conditions; however, the grain size of *GRH5*, *GRH2/GRH5*-PYL and *GRH2/GRH6*-PYL was smaller than in T65 (Table S4A).

During the screen house experiment, NILs including the PYLs generally attained a greater biomass and production (grain yield) than T65, with the highest yields on *GRH2/GRH4*-PYL, *GRH2/GRH5*-PYL and *GRH2/GRH4/GRH6*-PYL because of their high resistance to GLH infestations (Fig. 3 J–L, Table S4B). Significant nitrogen × infestation interactions for panicle and grain number were due to reductions in these yield components resulting from GLH feeding under all nitrogen regimes and significant line × infestation interactions were due to significant loses in plant production under GLH infestation in T65 and the three monogenic NILs, but with a maintenance of yield in some of the PYLs (Fig. 3 J–L, Table S4B). Nitrogen had no effect on proportional changes in shoot and root biomass between control and infested plants (shoot biomass: F_2,119_ = 0.022, P = 0.979; yield: F_2,119_ = 1.740, P = 0.236; Fig. 3; Tables S4A and S4B). Lines differed in their capacity to maintain growth and yield under the influence of herbivore pressure (shoot biomass: F_7,119_ = 4.392, P = 0.009; yield: F_7,119_ = 7.051, P = 0.001) with PYLs maintaining consistently higher production than NILs and *GRH2/GRH4/GRH6*-PYL showing no yield loses when infested with GLH (Fig. 3 P–R). Interaction terms were not significant.

When infested with GLH, the number of loci were positively correlated with shoot biomass (0 Kg N ha^−1^: C_8_ = 0.810, P = 0.015; 60 Kg N ha^−1^: C_8_ = 0.764, P = 0.027; 150 Kg N ha^−1^: C_8_ = 0.914, P = 0.002) and grain yield (0 Kg N ha^−1^: C_8_ = 0.773, P = 0.024; 60 Kg N ha^−1^: C_8_ = 0.813, P = 0.014; 150 Kg N ha^−1^: C_8_ = 0.923, P = 0.001) with increasingly consistent production of foliage (C_8_ = - 0.751, P = 0.032: Fig. 1 E) and grain (C_8_ = −0.856, P = 0.007: Fig. 1 F) on lines with higher numbers of genes. Similarly, in the greenhouse experiment, the biomass and yields of infested plants were often correlated with the numbers of genes in the lines (biomass 0 Kg N ha^−1^: C_8_ = 0.433, P = 0.248; 60 Kg N ha^−1^: C_8_ = 0.756, P = 0.004; 150 Kg N ha^−1^: C_8_ = 0.746, P = 0.005; yield 0 Kg N ha^−1^: C_8_ = 0.151, P = 0.639; 60 Kg N ha^−1^: C_8_ = 0.700, P = 0.011; 150 Kg N ha^−1^: C_8_ = 0.740, P = 0.006; see Tables S3A and S3B). The shoot biomass of control, non-infested plants was not correlated with the number of resistance loci under 0, 60 or 150 Kg N ha^−1^ (Fig. 1 G). However, grain yields were negatively correlated with the number of loci at 0 Kg N ha^−1^ (C_8_ = −0.720, P = 0.044) and 150 Kg N ha^−1^ (C_8_ = −0.010, P = 0.015), but not under 60 Kg N ha^−1^ (C_8_ = −0.249, P = 0.397: Fig. 1 H).

Nitrogen affected the number of panicles, number of grain per plant and the weight of grain in the field plot experiment (Table S5). There was no effect of rice line on any of the measured growth and yield parameters and no significant correlations between loci number and shoot biomass (0 Kg N ha^−1^: C_14_ = 0.028, P = 0.923; 60 Kg N ha^−1^: C_14_ = −0.130, P = 0.658) or yield (0 Kg N ha^−1^: C_14_ = −0.166, P = 0.570; 60 Kg N ha^−1^: C_14_ = −0.195, P = 0.504) (Table S5).

## 4 Discussion

Among the most effective lines against GLH in our study were *GRH2/GRH4*-PYL and *GRH2/GRH4/GRH6*-PYL, which reduced nymph survival, nymph growth, and damage to rice seedlings, and *GRH5/qGRH4*-PYL, which reduced nymph survival on early tillering plants. A number of pyramided lines (*GRH2/GRH4*-PYL, *GRH2/GRH5*-PYL, *GRH2/GRH6*-PYL, *GRH4/GRH6*-PYL and *GRH2/GRH4/GRH6*-PYL) as well as the monolocus line *qGRH4*-NIL effectively reduced oviposition in either seedlings or in both seedlings and early tillering plants. Two of these lines, *GRH2/GRH6*-PYL and *GRH2/GRH4/GRH6*-PYL, were associated with decreases in egg hatching rates. The greater effectiveness of PYLs compared to NILs in reducing egg-laying by multiple phloem-feeding species indicates the greater spectrum of resistance that can be achieved by combining two or more resistance loci in one plant. These results highlight the need for greater attention to the possible benefits of pyramiding in affecting herbivore species outside the species for which the loci were originally targeted (i.e., the species used during phenotyping and gene discovery). However, just as such broad approaches to resistance research will reveal unanticipated benefits, they may also indicate potential trade-offs. For example, our research indicated that, whereas *qGRH4* was associated with resistance against nymph feeding in GRH and decreased egg-laying in GLH, two lines with *qGRH4* in our study actually stimulated egg-laying in WBPH. Similar responses have been noted with GLH by Srinivasan et al. (2015) on a BPH-resistant pyramided line (*BPH25/BPH26*-PYL).

### 4.1 Resistance mechanisms

Gaps remain in our understanding of the actual resistance mechanisms expressed through many of the genes used in this study. Leafhoppers normally feed on rice phloem, but on resistant rice varieties they tend to feed more on xylem (ZLH: Choi et al., 1973, Pongprasert, 1974; GRH: Kawabe, 1985; GLH: Khan and Saxena, 1985). Xylem feeding is associated with increased probing, avoidance or non-preference of resistant varieties and resulting lower weight gains or increased mortality of nymphs or adults (Khan and Saxena, 1985). Leafhopper nymph mortality on resistant plants is frequently high. However, resistance against nymphs appears to be restricted to localized populations and is therefore not generally broad-spectrum (Heinrichs and Rapusas, 1985, 1990). Furthermore, adaptation by leafhoppers to antibiosis resistance is often rapid (Heinrichs and Rapusas, 1990; Vu et al., 2014; Horgan et al., 2018a).

Using DNA microarray analysis of rice plants (*GRH2*-NIL and *GRH2/GRH4*-PYL) during GRH infestation, Asano et al. (2015) indicated that volatiles play an important role in defending rice against leafhoppers. Their research revealed that *terpene synthase* was strongly and rapidly upregulated in *GRH2/GRH4*-PYL when compared to *GRH2*-NIL, indicating a strong induction of sesquiterpenes and allowing a more rapid defence response to GRH attack in the pyramided line. Whether these defences also act to reduce egg-laying in leafhoppers and planthoppers is unknown. Our results indicate a further effect of resistance on GLH populations. Although not statistically significant, two plants, each with the *GRH2* and *GRH6* loci (*GRH2/GRH6*-PYL and *GRH2/GRH4/GRH6*-PYL) had higher proportions of unhatched eggs that may indicate an ovicidal response. Ovicidal responses in *japonica* rice lines are normally strongest during late stages of plant growth (i.e., late tillering)(Horgan et al., 2016). These two PYLs could be evaluated at later growth stages to verify whether they cause higher rates of egg mortality.

### 4.2 Resistance strength

Several recent studies have evaluated the comparative strength of resistance in rice lines with one, two and/or three resistance loci (BPH: Myint et al., 2009, 2012; Li et al., 2011; Hu et al., 2012, 2015, 2016b; Srinivasan et al., 2015; Liu et al., 2016; Jena et al., 2017; Wang et al., 2017; Han et al., 2018; Jiang et al., 2018) including evaluations of some of the materials used in the present study (GRH: Fujita et al., 2010; GLH: Vu et al., 2014; Horgan et al., 2018a) (Table 2). In many of these studies, ‘resistance’ was compared using seedbox screening tests. Such tests have been criticized because they fail to distinguish antibiosis from tolerance (the plant's ability to compensate for damage) and are therefore not a true measure of resistance (Panda and Heinrichs, 1983; Velusamy et al., 1986; Fujita et al., 2013). Nevertheless, among the studies that directly assessed resistance, PYLs have been consistently more effective than monogenic NILs in reducing leafhopper or planthopper fitness (Table 2). Furthermore, PYLs with three loci had consistently stronger resistance that PYLs with two loci (Hu et al., 2012, 2016b; Jena et al., 2017, Table 2). Nevertheless, a true test of the hypothesis that pyramiding loci increases resistance is to show that the number of loci in a variety is correlated with resistance strength across all possible combinations of loci and irrespective of the genes involved. Our study with GLH revealed a significant effect of loci number on resistance strength measured as decreased egg-laying and nymph biomass (Fig. 1A and B). Our results therefore support the hypothesis that increasing the numbers of resistance genes/loci produces stronger resistance against phloem-feeding herbivores.

### 4.3 Resistance spectrum

Pyramiding resistance genes is purported to produce broad-spectrum resistance (Hu et al., 2016b; Fan et al., 2017). This relationship is seldom evaluated in studies with resistant rice because most studies limit their experiments to a single laboratory colony of the target herbivore (Table 2). To assess the spectrum of resistance, more than one herbivore population is required and these populations should respond differentially to the range of test lines (i.e., the populations should represent different ‘biotypes’). Hirae et al. (2007) were among the first to indicate that rice lines with pyramided resistance genes can have a greater spectrum of resistance than related monogenic lines. These authors produced three ‘biotypes’ of GRH through selective breeding and tested these on varieties (not near-isogenic lines) with one or two resistance genes. Their results indicated that lines with two genes affected more of the biotypes than lines with one gene.

Only two previous studies have used near-isogenic rice lines to support the hypothesis that pyramiding increases the resistance spectrum of rice against leafhoppers or planthoppers. Myint et al. (2009) indicated that a *BPH25/BPH26-*PYL was resistant to four colonies of BPH, but that each of the corresponding monogenic NILs were resistant to only two of the colonies. Jena et al. (2017) conducted feeding bioassays (honeydew excretion tests) with four populations of BPH from the Philippines using an extensive collection of NILs of which several were multi-loci PYLs. Their results indicate that three of the populations had similar patterns of response across the range of lines, but one population (from Isabella, Philippines) appeared largely virulent against four of the monogenic NILs.

The spectrum of resistance has generally only been considered within a single herbivore species (but see Srinivasan et al., 2015); however, in our study we examined the responses by a range of phloem-feeding leafhoppers and planthoppers to the 14 rice lines. To test the hypothesis, we compared the proportions of herbivores showing reductions in fitness on the lines against the numbers of genes in the lines. We found that by increasing the number of resistance genes/loci in the near-isogenic lines, nymph survival and adult egg-laying were reduced in increasingly larger numbers of hopper species (Fig. 1C and D). These results are novel only because phenotyping, gene discovery and research on resistance mechanisms are normally restricted to a single herbivore species, with resulting genes/loci named according to that species (e.g., BPH, GLH, WBPH) without acknowledging the broader potential of the genes either alone, or when combined with other resistance genes. As indicated in our study, identified loci can have similar effects on two or more species, or impart novel resistance traits when pyramided. Loci with resistance for one species may also increase susceptibility to a different herbivore species, as in the case of *qGRH4* and WBPH. Therefore, as indicated in our study, because the mechanisms of resistance associated with specific genes are still largely unknown (see section 4.1), the effects of pyramiding resistance genes can bring unanticipated outcomes for other herbivores. Our results therefore strongly support the hypothesis that increasing the numbers of resistance genes/loci will generally result in resistance against a broader spectrum of phloem-feeding herbivores.

### 4.4 Resistance stability

Stability is a key factor in the success of resistance. For example, Jairin et al. (2007) indicated that BPH affected by the resistance of Rathu Heenati (*BPH3*/*BPH32*) in Thailand could still feed on the variety's rice panicles at the time of grain filling. Vu et al. (2014) evaluated the stability of *GRH2*-NIL, *GRH4*-NIL and *GRH2/GRH4*-PYL as these plants aged. Their results indicated that the PYL was stable throughout plant development (albeit with severe reductions in yield resulting from heavy infestations during the seedling stage). In contrast, the *GRH4*-NIL was moderately resistant only at mid-tillering (45 DAS), but not at other plant stages.

In the present study, we also assessed the resistance stability of NILs under gradients of nitrogen fertilizer. On infested *GRH2/GRH5*-PYL and *GRH2/GRH4/GRH6*-PYL the biomass of GLH was consistently low under all fertilizer regimes (Fig. 3G–I), indicating a greater stability of resistance in both these lines compared to the NILs and other PYLs. Many of the 2-loci PYLs and *GRH2/GRH4/GRH6*-PYL maintained their capacity to respond to increasing nitrogen levels through increased shoot growth despite GLH infestation. In contrast, only the three-loci PYL could respond to added nitrogen by increasing yield. These results indicate that the greater stability of yields in PYLs with two or three loci (Fig. 1E and F) were due, not only to stable resistance, but also to a component of tolerance that may have been associated with some of the loci. Because we only examined a single 3-loci PYL, but six two-loci PYLs (four of which were resistant to GLH), our support for a general hypothesis about pyramiding and resistance stability is not strong. However, the results do indicate that resistance and tolerance in the 3-loci PYL were the most stable from among resistant lines in our materials.

### 4.5 Resistance durability

The principal aim of pyramiding has been to increase the durability of resistance in the field. Indeed, pyramiding has primarily been a response to the rapid breakdown of *BPH1* and *BPH2* in the 1980s and 1990s and the emergence of planthopper populations with virulence against *BPH3* and *BPH4* in South East Asia and China (Horgan, 2018). Despite this objective, few studies have compared leafhopper or planthopper virulence adaptation on polygenic and monogenic rice lines. Ferrater et al. (2015) found that BPH populations from six sites in the Philippines adapted to IR62 (*BPH3* and/or *BPH32*) and IR65482-4-136-2-2 (*BPH10*) within 12–15 generations, but the polygenic variety PTB33 (*BPH2*, *BPH3* and/or *BPH32*, and *ZLH3*) remained resistant for over 20 generations. Similarly, Hirae et al. (2007) found that GRH failed to establish and adapt to Norin-PL5 (*GRH2* and *GRH4*) and Norin-PL6 (*GRH2* and *GRH4*), whereas populations virulent against the monogenic line Kanto-PL6 could be selected within five to eight generations.

We did not examine resistance durability in the present study; however, evidence from a related paper, Horgan et al. (2018a) indicated that the resistance of *GRH2/GRH4*-PYL to GLH in selection experiments has been more durable than monolocus resistance evaluated in similar experiments (Heinrichs and Rapusas, 1990; Dahal et al., 1997). However, the same study demonstrated that adaptation to monogenic NILs (*GRH2-*NIL and *GRH4-*NIL) compromised pyramided resistance. This suggests that the field durability of PYLs is ultimately determined by the nature of the genes in the line. Future studies should take a holistic approach to evaluating resistance durability to test whether pyramided lines provide field resistance against more generations of leafhoppers than would the sequential deployment of each of the genes in the pyramided line. The availability of improved research materials such as NILs that share a recurrent parental lines should encourage future experiments to compare selection rates and answer one of the principal questions concerning the deployment of resistance genes.

### 4.6 Potential trade-offs

Ecological costs to the host plant of strong resistance can be manifested in many ways. Principally these will include reductions in growth, reproduction or maintenance – including reduced tolerance to biotic and abiotic stresses (Herms and Mattson, 1992; Brown, 2002; Horgan et al., 2013). In rice, potential trade-offs between defence and reproductive output (grain yield) is a key consideration for the success of resistance breeding. However, few studies have examined these trade-offs in any detail. A number of studies with transgenic rice crops indicate that the transgenes may reduce yields, but that resistance results in higher yields under high herbivore pressure (Chen et al., 2006; Xia et al., 2010; Kim et al., 2008; Wang et al., 2012).

A number of studies that examined the agronomic traits of rice NILs in field plots have reported generally higher or similar yields to the recurrent parent lines (Table 2) or report generally similar agronomic traits, without comparing yields (i.e., Jena et al., 2017). It is difficult to understand how yields could increase when introgressed with traits derived from traditional varieties or wild rice species. However, studies indicating yield increases often omit key aspects of field management, including the possible use of resurgence-causing insecticides, and do not report insect pressures in the field (Table 2). For example, using a range of NILs including some PYLs, Liu et al. (2016) indicated that resistant lines with *BPH3* and *BPH2*7 had generally lower yields that the recurrent parents, Ningjing3 and 93-11, under insect-free conditions, but had higher yields when exposed to natural herbivore pressures. Similarly, in the present study, we noted significant yield penalties associated with pyramiding resistance loci on plants grown without herbivore pressure. These were most pronounced in the three-loci PYL and were generally consistent across gradients of nitrogen application (Fig. 1G and H).

When infested with GLH, as expected, the three-loci PYL performed the best and maintained or increased (overcompensated) predicted yields based on control plants. When grown in rice paddies and exposed to natural herbivory, there was no apparent relation between yields and the numbers of genes in the lines; however, the improved lines rarely produced more than the recurrent parent T65. Although our results indicate that increasing the number of resistance genes/loci has associated ecological costs, the hypothesis is not universally supported (e.g., Wiarda et al., 2012; McCarville et al., 2014). Furthermore, without examining potential yield penalties, several studies with genes introgressed into hybrid rice varieties have achieved high yielding plants despite apparently strong resistance from two (Xu, 2013; Fan et al., 2017; Hu et al., 2012, 2016b; Xiao et al., 2016) or three (Hu et al., 2016b) loci/genes. It is probable that robust hybrid plants have a greater capacity to compensate for expensive defences in a manner analogous to tolerance against herbivores and diseases. In comparisons of crops with two, three and four loci, resistance is often sufficiently strong with two genes such that increasing the number of genes further has no real advantage for production (Zhang et al., 2018). This suggests that the optimal number of genes for deployment in pyramided lines will be the smallest number that achieves effective and durable resistance.

### 4.7 Concluding remarks

Given the large research investments in developing resistant rice for Asia, the history of virulence adaptations and the effective loss of rare resistance genes, there has been a surprising lack of research that specifically addresses the claimed benefits of pyramiding. It is also important to remember that outbreaks and yield losses from many of the key phloem feeders of rice are a direct result of poor crop management, particularly the overuse of insecticides (Bottrell and Schoenly, 2012; Horgan and Crisol, 2013). It is imperative that improved resistance does not simply mask the consequences of indiscriminate insecticide use. As resistance breeding continues, research would greatly benefit by more directly addressing ecological and evolutionary theories when assessing host-herbivore interactions, and the influence of environment on these interactions, to avoid future problems of virulence adaptation. Further environmental and sociological research is therefore also required to take best advantage of emerging resistant varieties and the increased availability of near-isogenic lines with resistance genes, to develop truly sustainable rice productions systems.

## Appendix A Supplementary data

GLH pyramiding 02 acceptedchanges - Horgan et al.docx [42–58] GLH pyramiding 02 acceptedchanges - Horgan et al.docx [42–58]
