# Supplementary material for: Unanticipated benefits and potential ecological costs associated with pyramiding leafhopper resistance loci in rice
Source: Crop Prot. 2019 Jan;115:47–58. doi: 10.1016/j.cropro.2018.09.013 (PMC6358143; doi:10.1016/j.cropro.2018.09.013)
Supplement: GLH pyramiding 02 acceptedchanges - Horgan et al.docx [42–58] [file mmc1.docx]

**Table S1**: Results of greenhouse bioassay with *Nilaparvata lugens* on 14 near-isogenic rice lines with zero, one, two or three resistance loci.

| Line^a^ | Eggs laid^b^ | Nymph weight (mg)^b^ | Nymph development^c^ | |  |
| --- | --- | --- | --- | --- | --- |
|  |  |  | Ist instar | 2nd instar | 3rd instar |
| T65 | 104.67±13.85 | 1.56±0.23 | 0.65±0.06 | 0.24±0.03 | 0.10±0.04 |
| IR24 (*GRH1*) | 144.83±14.06 | 3.26±0.66 | 0.60±0.08 | 0.25±0.03 | 0.15±0.05 |
| *GRH1-NIL* | 105.17±11.72 | 1.48±0.25 | 0.62±0.05 | 0.31±0.05 | 0.07±0.04 |
| *GRH2-NIL* | 109.50±17.61 | 2.12±0.27 | 0.61±0.06 | 0.29±0.03 | 0.11±0.03 |
| *GRH4-NIL* | 88.17±20.20 | 1.75±0.26 | 0.62±0.10 | 0.21±0.04 | 0.17±0.06 |
| *GRH5-NIL* | 100.83±13.40 | 1.64±0.36 | 0.68±0.04 | 0.25±0.05 | 0.07±0.02 |
| *GRH6-NIL* | 91.00±5.09 | 1.47±0.25 | 0.59±0.08 | 0.36±0.08 | 0.04±0.03 |
| *qGRH4-NIL* | 115.00±14.74 | 2.31±0.41 | 0.48±0.05 | 0.39±0.07 | 0.13±0.03 |
| *GRH2/GRH4*-PYL | 200.00±19.07 | 2.69±0.19 | 0.75±0.06 | 0.18±0.04 | 0.07±0.02 |
| *GRH2/GRH5*-PYL | 92.67±19.15 | 1.38±0.24 | 0.68±0.11 | 0.26±0.10 | 0.06±0.02 |
| *GRH2/6GRH*-PYL | 43.20±5.84* | 0.81±0.15 | 0.76±0.08 | 0.18±0.07 | 0.06±0.02 |
| *GRH4/GRH5*-PYL | 95.50±7.36 | 1.61±0.16 | 0.70±0.06 | 0.26±0.04 | 0.04±0.03 |
| *GRH4/GRH6*-PYL | 64.50±8.95 | 1.07±0.21 | 0.59±0.05 | 0.34±0.04 | 0.07±0.03 |
| *GRH5/qGRH4*-PYL | 118.33±19.46 | 2.06±0.52 | 0.76±0.06 | 0.18±0.04 | 0.06±0.02 |
| *GRH2/GRH4/GRH6*-PYL | 36.67±9.32** | 0.60±0.17* | 0.68±0.07 | 0.25±0.06 | 0.07±0.03 |
| F_14,75_-value | 7.596*** | 5.373*** | 1.181ns | 1.321ns | 1.203ns |

a: IR24 containing the *GRH1* gene was included as an *indica* check variety and is not isogenic with T65

b: Numbers are means ± SEM (N = 5); *** = P ≤ 0.001, ** = P ≤ 0.01, * = P ≤ 0.05, ns = P ≥ 0.05; asterisks adjacent to SEMs are based on Duncan’s many-to-one comparisons.

c: Numbers are mean proportions ± SEM; Data arcsine transformed; F-values are for between subject effects

**Table S2A**: Results of insectary bioassays with four herbivore species on 14 near-isogenic rice lines with zero, one, two or three resistance loci. All herbivore species were rice phloem-feeders. These were the green leafhopper (GLH), zig-zag leafhopper (ZLH), brown planthopper (BPH) and whitebacked planthopper (WBPH). See also Table S2B.

| Lines | Days to wilt^a^ |  |  |  | Eggs laid^a^ |  |  |  |
| --- | --- | --- | --- | --- | --- | --- | --- | --- |
|  | GLH | ZLH | BPH | WBPH | GLH | ZLH | BPH | WBPH |
| T65 | 8.20±0.40 | 7.00±0.00 | 7.00±0.00 | 6.67±0.33 | 104.40±13.85 | 12.50±2.09 | 104.67±13.85 | 98.83±8.05 |
| *GRH1-NIL* | 9.00±0.00 | 7.00±0.00 | 6.67±0.33 | 7.00±0.00 | 120.60±17.33 | 25.67±3.46 | 105.17±11.72 | 109.83±12.63 |
| *GRH2-NIL* | 9.00±0.00 | 6.33±0.67 | 7.00±0.00 | 7.00±0.00 | 80.40±11.72 | 18.17±3.74 | 109.50±17.61 | 49.17±10.14** |
| *GRH4-NIL* | 9.00±0.00 | 7.00±0.00 | 6.67±0.33 | 7.00±0.00 | 92.60±22.33 | 6.17±1.17* | 88.17±20.20 | 98.00±12.36 |
| *GRH5-NIL* | 9.00±0.00 | 7.00±0.00 | 7.00±0.00 | 7.00±0.00 | 153.00±20.42 | 14.00±1.98 | 100.83±13.40 | 57.67±11.01 |
| *GRH6-NIL* | 10.20±0.40** | 7.00±0.00 | 6.67±0.33 | 7.00±0.00 | 58.40±7.81 | 7.50±1.20 | 91.00±5.09 | 79.67±8.67 |
| *qGRH4-NIL* | 8.20±0.83 | 7.00±0.00 | 6.00±0.45 | 7.00±0.00 | 33.80±8.12*** | 20.67±2.86 | 96.83±23.40 | 247.17±32.70*** |
| *GRH2/GRH4-PYL* | 15.00±0.00*** | 7.00±0.00 | 7.00±0.00 | 7.00±0.00 | 30.40±4.85*** | 14.17±2.64 | 142.00±5.60 | 100.83±11.36 |
| *GRH2/GRH5-PYL* | 11.00±0.52*** | 7.00±0.00 | 6.33±0.67 | 7.00±0.00 | 41.50±7.31** | 18.33±2.09 | 92.67±19.15 | 73.50±14.29 |
| *GRH2/GRH6-PYL* | 9.00±0.52 | 7.00±0.00 | 6.67±0.33 | 7.00±0.00 | 45.60±4.30* | 9.40±2.89 | 36.33±9.01*** | 25.67±4.51*** |
| *GRH4/GRH5-PYL* | 8.60±0.33 | 7.00±0.00 | 7.00±0.00 | 7.00±0.00 | 93.00±14.90 | 8.50±1.80 | 95.50±7.36 | 116.33±22.61 |
| *GRH4/GRH6-PYL* | 9.80±0.40 | 7.00±0.00 | 6.33±0.42 | 7.00±0.00 | 55.00±8.92* | 13.17±2.95 | 64.50±8.95 | 36.17±6.34*** |
| *GRH5/qGRH4-PYL* | 8.20±0.83 | 7.00±0.00 | 7.00±0.00 | 7.00±0.00 | 78.20±4.69 | 14.17±3.68 | 118.33±19.46 | 193.00±31.81*** |
| *GRH2/GRH4/GRH6-PYL* | 15.00±0.00*** | 7.00±0.00 | 6.67±0.33 | 7.00±0.00 | 28.00±2.80*** | 13.50±1.77 | 36.67±9.32*** | 20.50±3.84*** |
| F_13,70_-value | 29.092*** | 1.000ns | 1.017ns | 1.000ns | 8.451*** | 4.232*** | 6.243*** | 15.591*** |

a: Numbers are means ± SEM (N = 6); *** = P ≤ 0.001, ** = P ≤ 0.01, * = P ≤ 0.05, ns = P ≥ 0.05; asterisks adjacent to SEMs are based on Duncan’s many-to-one comparisons. Underlined asterisks indicate an increase in egg-laying by the whitebacked planthopper.

**Table S2B:** Results of insectary bioassays with four herbivore species on 14 near-isogenic rice lines with zero, one, two or three resistance loci. All herbivore species were rice phloem-feeders. These were the green leafhopper (GLH), zig-zag leafhopper (ZLH), brown planthopper (BPH) and whitebacked planthopper (WBPH). See also Table S2 A.

| Line | Nymph survival (proportion) | |  |  | Nymph biomass (mg) | |  |  |
| --- | --- | --- | --- | --- | --- | --- | --- | --- |
|  | GLH | ZLH | BPH | WBPH | GLH | ZLH | BPH | WBPH |
| T65 | 0.95±0.03 | 0.96±0.03 | 0.98±0.02 | 0.90±0.04 | 0.24±0.01 | 0.20±0.02 | 0.23±0.02 | 0.13±0.02 |
| *GRH1-NIL* | 0.95±0.03 | 0.94±0.04 | 1.00±0.00 | 0.85±0.02 | 0.25±0.01 | 0.22±0.02 | 0.23±0.02 | 0.12±0.01 |
| *GRH2-NIL* | 0.90±0.06 | 0.96±0.04 | 1.00±0.00 | 0.88±0.05 | 0.26±0.02 | 0.22±0.02 | 0.23±0.02 | 0.12±0.01 |
| *GRH4-NIL* | 0.93±0.04 | 0.98±0.02 | 0.96±0.04 | 0.85±0.06 | 0.28±0.01 | 0.23±0.02 | 0.22±0.02 | 0.12±0.02 |
| *GRH5-NIL* | 0.93±0.03 | 0.98±0.02 | 1.00±0.00 | 0.85±0.02 | 0.21±0.01 | 0.23±0.02 | 0.23±0.02 | 0.12±0.01 |
| *GRH6-NIL* | 0.93±0.04 | 0.96±0.03 | 0.92±0.06 | 0.81±0.09 | 0.30±0.01 | 0.22±0.02 | 0.23±0.03 | 0.11±0.02 |
| *qGRH4-NIL* | 0.85±0.05 | 0.96±0.04 | 0.96±0.04 | 0.91±0.05 | 0.22±0.01 | 0.22±0.01 | 0.22±0.02 | 0.13±0.02 |
| *GRH2/GRH4-PYL* | 0.31±0.09*** | 0.94±0.06 | 0.92±0.06 | 0.81±0.04 | 0.09±0.03*** | 0.22±0.02 | 0.23±0.03 | 0.11±0.02 |
| *GRH2/GRH5-PYL* | 0.78±0.08 | 1.00±0.00 | 0.96±0.04 | 0.96±0.03 | 0.22±0.02 | 0.23±0.02 | 0.23±0.02 | 0.13±0.02 |
| *GRH2/GRH6-PYL* | 0.95±0.03 | 0.92±0.04 | 1.00±0.00 | 0.81±0.03 | 0.28±0.01 | 0.21±0.02 | 0.23±0.02 | 0.11±0.02 |
| *GRH4/GRH5-PYL* | 0.95±0.04 | 0.96±0.04 | 0.98±0.02 | 0.88±0.05 | 0.33±0.01 | 0.22±0.02 | 0.23±0.02 | 0.12±0.01 |
| *GRH4/GRH6-PYL* | 0.98±0.02 | 0.92±0.04 | 0.98±0.02 | 0.92±0.04 | 0.31±0.01 | 0.21±0.02 | 0.23±0.02 | 0.13±0.02 |
| *GRH5/qGRH4-PYL* | 0.72±0.07 | 0.96±0.03 | 1.00±0.00 | 0.94±0.04 | 0.23±0.02 | 0.22±0.02 | 0.23±0.02 | 0.13±0.02 |
| *GRH2/GRH4/GRH6-PYL* | 0.14±0.07*** | 0.98±0.02 | 0.94±0.06 | 0.85±0.06 | 0.05±0.03*** | 0.22±0.01 | 0.21±0.01 | 0.12±0.02 |
| F_13,70_-value | 11.154*** | 0.495ns | 0.695ns | 1.304ns | 24.609*** | 0.155ns | 0.089ns | 0.131ns |

a: Numbers are means ± SEM (N = 6); *** = P ≤ 0.001, ns = P ≥ 0.05; asterisks adjacent to SEMs are based on Duncan’s many-to-one comparisons.

**Table S3A**: Results from greenhouse experiment with 12 lines under three nitrogenous fertilizer regimes (equivalent to 0, 60 and 150 Kg N ha^-1^). Plants were not exposed to insect herbivores. See Table S3 B for results from infested plants. The experimental protocol is outlined below Table S3B.

| Line | Root dry weight (g) | Number of tillers | Proportion of tillers productive | Shoot dry weight (g) | Number of panicles | Number of grain | Proportion of grain filled | Weight of filled grain (g) |
| --- | --- | --- | --- | --- | --- | --- | --- | --- |
| Zero added nitrogen |  | A |  | A | A | A |  |  |
| T65 | 7.48±1.08 | 10.60±0.60abc | 0.66±0.10ab | 23.79±1.08ab | 6.00±0.32bc | 485.60±56.99abc | 0.54±0.06c | 6.26±1.38bc |
| *GRH1-NIL* | 9.42±2.00 | 8.60±1.54ab | 0.65±0.14ab | 26.41±2.37ab | 4.40±0.24ab | 525.40±56.95ab | 0.38±0.08bc | 6.09±1.38abc |
| *GRH2-NIL* | 6.98±2.75 | 8.60±1.40abc | 0.60±0.04ab | 23.16±2.65b | 4.80±0.66bc | 451.40±47.52abcd | 0.54±0.10c | 5.68±1.50bc |
| *GRH4-NIL* | 7.21±1.10 | 10.80±0.80bc | 0.60±0.03ab | 26.25±3.31ab | 7.00±0.84bc | 589.80±82.32abcd | 0.61±0.13c | 7.79±2.19bc |
| *GRH5-NIL* | 6.94±0.80 | 5.80±0.66a | 0.86±0.07b | 23.14±3.39a | 5.00±0.71ab | 513.40±61.03ab | 0.56±0.10c | 7.05±1.83abc |
| *GRH6-NIL* | 8.99±2.71 | 11.00±0.71c | 0.54±0.07a | 19.76±2.70ab | 5.25±0.97bc | 761.40±167.97d | 0.23±0.08ab | 4.65±1.92abc |
| *GRH2/GRH4-PYL* | 4.00±1.24 | 6.80±2.29ab | 0.78±0.14ab | 17.71±5.07ab | 4.00±1.10a | 279.80±94.79a | 0.48±0.14bc | 3.19±1.77ab |
| *GRH2/GRH5-PYL* | 6.91±1.19 | 10.25±0.86bc | 0.64±0.07ab | 24.40±1.83ab | 5.75±0.49abc | 490.60±47.52ab | 0.17±0.07a | 1.81±0.88a |
| *GRH2/GRH6-PYL* | 8.29±1.59 | 9.25±0.66bc | 0.74±0.10ab | 25.10±2.80b | 5.25±0.58abc | 636.00±100.50bcd | 0.33±0.12abc | 5.10±2.05abc |
| *GRH4/GRH5-PYL* | 8.82±2.34 | 10.50±2.29c | 0.77±0.10ab | 22.09±3.62b | 6.75±1.02d | 580.50±123.53cd | 0.73±0.03c | 9.22±2.56c |
| *GRH4/GRH6-PYL* | 8.08±1.52 | 11.20±0.86bc | 0.63±0.10ab | 23.98±1.03ab | 6.60±0.60bc | 609.00±106.04abc | 0.45±0.12bc | 6.90±2.73bc |
| *GRH2/GRH4/GRH6-PYL* | 6.05±0.52 | 10.80±1.77bc | 0.73±0.06ab | 24.66±3.13ab | 6.00±0.84bc | 506.00±50.45abcd | 0.44±0.13bc | 4.93±1.63bc |
| 60 Kg N ha^-1^ |  | B |  | B | B | AB |  |  |
| T65 | 9.41±1.37 | 11.80±1.39 | 0.79±0.06 | 29.50±1.76 | 7.60±1.03 | 558.80±86.73 | 0.52±0.08 | 6.19±1.30 |
| *GRH1-NIL* | 12.88±3.24 | 10.00±1.38 | 0.74±0.07 | 29.35±2.49 | 6.60±0.68 | 579.20±75.70 | 0.58±0.10 | 9.08±2.46 |
| *GRH2-NIL* | 7.89±1.53 | 12.80±1.53 | 0.70±0.06 | 34.93±2.82 | 8.00±1.00 | 618.00±106.80 | 0.41±0.13 | 6.70±2.85 |
| *GRH4-NIL* | 7.21±1.67 | 13.40±1.83 | 0.60±0.03 | 26.90±1.63 | 7.40±0.75 | 530.80±84.12 | 0.69±0.10 | 7.96±2.07 |
| *GRH5-NIL* | 5.91±0.57 | 6.80±0.80 | 0.89±0.03 | 16.24±3.98 | 5.00±0.45 | 360.20±66.16 | 0.57±0.07 | 4.71±1.17 |
| *GRH6-NIL* | 11.04±4.45 | 16.40±2.09 | 0.58±0.05 | 29.88±1.18 | 9.20±0.49 | 886.60±79.42 | 0.36±0.08 | 6.76±2.06 |
| *GRH2/GRH4-PYL* | 6.26±0.82 | 9.80±0.58 | 0.67±0.02 | 25.69±2.33 | 4.80±058 | 320.80±31.19 | 0.42±0.05 | 2.53±0.45 |
| *GRH2/GRH5-PYL* | 4.66±0.57 | 14.00±1.26 | 0.54±0.03 | 29.22±2.60 | 6.25±0.43 | 375.75±52.41 | 0.05±0.04 | 0.53±0.45 |
| *GRH2/GRH6-PYL* | 7.91±1.56 | 14.40±2.04 | 0.62±0.09 | 30.83±1.91 | 8.20±0.58 | 577.60±109.43 | 0.36±0.09 | 4.89±1.99 |
| *GRH4/GRH5-PYL* | 9.48±1.80 | 17.20±1.11 | 0.67±0.06 | 32.87±1.92 | 11.40±1.03 | 803.20±78.14 | 0.62±0.04 | 9.85±1.45 |
| *GRH4/GRH6-PYL* | 6.12±0.81 | 11.20±0.66 | 0.72±0.06 | 30.21±2.47 | 7.20±0.92 | 474.40±46.07 | 0.44±0.19 | 7.89±0.29 |
| *GRH2/GRH4/GRH6-PYL* | 8.73±0.88 | 14.00±0.71 | 0.63±0.01 | 29.73±2.33 | 8.20±0.66 | 686.80±56.83 | 0.55±0.05 | 5.05±2.21 |
| 150 Kg N ha^-1^ |  | B |  | B | C | B |  |  |
| T65 | 7.84±1.92 | 11.20±1.74 | 0.80±0.06 | 30.88±4.84 | 8.60±0.81 | 644.40±111.96 | 0.63±0.12 | 9.69±2.55 |
| *GRH1-NIL* | 5.19±1.22 | 10.20±1.20 | 0.67±0.04 | 28.74±3.79 | 5.80±1.39 | 307.80±97.42 | 0.31±0.10 | 3.02±1.32 |
| *GRH2-NIL* | 8.55±1.73 | 12.00±1.58 | 0.76±0.05 | 32.34±2.49 | 9.00±0.89 | 742.40±62.70 | 0.70±0.09 | 11.66±1.81 |
| *GRH4-NIL* | 12.48±4.47 | 14.00±0.63 | 0.74±0.05 | 27.37±4.70 | 9.60±0.24 | 657.40±39.63 | 0.50±0.07 | 6.64±1.63 |
| *GRH5-NIL* | 8.49±1.92 | 11.00±1.90 | 0.72±0.05 | 25.29±3.94 | 6.80±0.97 | 478.60±70.97 | 0.51±0.14 | 5.77±1.72 |
| *GRH6-NIL* | 5.73±1.09 | 16.20±1.53 | 0.60±0.03 | 31.59±1.27 | 9.40±0.51 | 758.80±105.97 | 0.26±0.09 | 4.16±1.84 |
| *GRH2/GRH4-PYL* | 6.43±1.09 | 12.20±1.36 | 0.68±0.10 | 29.68±1.82 | 6.80±0.49 | 570.60±113.37 | 0.42±0.10 | 4.84±1.13 |
| *GRH2/GRH5-PYL* | 5.17±1.34 | 12.25±2.23 | 0.70±0.11 | 32.05±6.78 | 6.50±1.18 | 600.50±136.67 | 0.25±0.11 | 2.78±1.09 |
| *GRH2/GRH6-PYL* | 6.54±1.75 | 12.00±1.38 | 0.70±0.03 | 32.95±2.89 | 7.60±1.40 | 706.20±156.21 | 0.46±0.12 | 7.93±2.63 |
| *GRH4/GRH5-PYL* | 9.66±0.93 | 16.20±2.33 | 0.78±0.09 | 41.03±5.04 | 11.80±0.86 | 941.00±50.70 | 0.49±0.05 | 9.68±1.25 |
| *GRH4/GRH6-PYL* | 8.75±1.67 | 12.60±2.01 | 0.87±0.07 | 29.06±2.78 | 9.60±0.51 | 616.60±103.14 | 0.58±0.07 | 7.57±1.54 |
| *GRH2/GRH4/GRH6-PYL* | 5.74±1.22 | 14.00±0.55 | 0.60±0.04 | 32.36±1.39 | 7.60±0.75 | 608.80±87.34 | 0.56±0.05 | 7.61±1.71 |
| F_11,140_-Line (L)^b^ | 1.558ns | 6.248*** | 2.599*** | 2.572*** | 10.377*** | 6.353*** | 6.887*** | 4.594*** |
| F_2,140_-Nitrogen (N)^b^ | 0.536ns | 21.849*** | 1.060ns | 22.105*** | 45.063*** | 4.515** | 0.150ns | 1.289ns |
| F_22,140_-L×N^b^ | 1.232ns | 0.911ns | 1.096ns | 4.180ns | 1.703* | 1.552ns | 1.202ns | 1.103ns |

a: Numbers are means ± SEM (N = 5); Upper case letters indicate homogenous fertilizer groups, lower case letters indicate homogenous line groups based on Tukey tests (P ≤ 0.05).

b: *** = P ≤ 0.001, ** = P ≤ 0.01, * = P ≤ 0.05, ns = P ≥ 0.05.

**Table S3B:** Results from greenhouse experiment with 12 lines under three nitrogenous fertilizer regimes (equivalent to 0, 60 and 150 Kg N ha^-1^). Plants were infested with four gravid female *Nephotettix virescens* (GLH) at 20 days after sowing^a^. See Table S3 A for results from non-infested, control plants.

| Line | GLH biomass density  (mg/g plant) | Root dry weight (g) | Number of tillers | Proportion of tillers productive | Shoot dry weight (g) | Number of panicles | Number of grain | Proportion of grain filled | Weight of filled grain (g) |
| --- | --- | --- | --- | --- | --- | --- | --- | --- | --- |
| Zero added nitrogen | A |  | A |  | A | A |  |  |  |
| T65 | 14.81±8.58c | 2.89±2.89 | 2.20±2.20abc | 0.55 | 7.54±5.57abc | 1.20±1.20abc | 152.60±152.60abc | 0.73 | 2.61±2.61bc |
| *GRH1-NIL* | 16.26±7.02abc | 2.55±2.55 | 2.20±2.20ab | 0.45 | 5.56±4.56abc | 1.00±1.00a | 135.60±135.60ab | 0.53 | 1.82±1.82abc |
| *GRH2-NIL* | 8.43±2.64ab | 0.00±0.00 | 0.00±0.00ab | - | 1.13±0.22abc | 0.00±0.00ab | 0.00±0.00abc | - | 0.00±0.00bc |
| *GRH4-NIL* | 13.52±3.39abc | 0.00±0.00 | 0.00±0.00abc | - | 1.08±0.23ab | 0.00±0.00abc | 0.00±0.00abc | - | 0.00±0.00abc |
| *GRH5-NIL* | 5.99±2.77abc | 0.00±0.00 | 0.00±0.00a | - | 0.87±0.10a | 0.00±0.00a | 0.00±0.00a | - | 0.00±0.00ab |
| *GRH6-NIL* | 5.56±2.25abc | 0.12±0.12 | 0.00±0.00bc | - | 2.27±0.67ab | 0.00±0.00abc | 0.00±0.00cd | - | 0.00±0.00ab |
| *GRH2/GRH4-PYL* | 2.09±0.96ab | 3.16±0.68 | 5.80±0.97bc | 0.74±0.09 | 14.68±2.02bc | 3.20±0.49abc | 250.20±62.35abc | 0.51±0.05 | 2.51±0.66ab |
| *GRH2/GRH5-PYL* | 12.79±5.52ab | 1.14±0.48 | 2.60±1.94cd | 0.58±0.08 | 7.28±3.23cd | 1.40±0.98abc | 89.40±68.37abc | 0.30±0.13 | 0.38±0.25a |
| *GRH2/GRH6-PYL* | 19.72±3.27abc | 0.31±0.20 | 0.00±0.00bc | - | 2.95±0.52bc | 0.00±0.00abc | 0.00±0.00abc | - | 0.00±0.00ab |
| *GRH4/GRH5-PYL* | 19.35±6.99bc | 0.00±0.00 | 0.00±0.00bc | - | 1.28±0.14bc | 0.00±0.00c | 0.00±0.00bcd | - | 0.00±0.00bc |
| *GRH4/GRH6-PYL* | 6.00±2.32ab | 0.00±0.00 | 0.00±0.00ab | - | 2.01±0.27ab | 0.00±0.00abc | 0.00±0.00abc | - | 0.00±0.00abc |
| *GRH2/GRH4/GRH6-PYL* | 4.75±1.61a | 2.81±0.69 | 5.60±1.21d | 0.83±0.08 | 14.49±2.76d | 4.00±0.77d | 368.20±76.11d | 0.44±0.11 | 4.11±1.44c |
| 60 Kg N ha^-1^ | A |  | B |  | B | B |  |  |  |
| T65 | 27.27±8.33 | 0.00±0.00 | 0.00±0.00 | - | 1.22±0.19 | 0.00±0.00 | 0.00±0.00 | - | 0.00±0.00 |
| *GRH1-NIL* | 3.88±1.61 | 0.00±0.00 | 0.00±0.00 | - | 1.17±0.17 | 0.00±0.00 | 0.00±0.00 | - | 0.00±0.00 |
| *GRH2-NIL* | 7.11±3.00 | 0.00±0.00 | 0.00±0.00 | - | 1.44±0.17 | 0.00±0.00 | 0.00±0.00 | - | 0.00±0.00 |
| *GRH4-NIL* | 11.00±3.16 | 0.00±0.00 | 0.00±0.00 | - | 1.04±0.20 | 0.00±0.00 | 0.00±0.00 | - | 0.00±0.00 |
| *GRH5-NIL* | 20.02±8.36 | 0.00±0.00 | 0.00±0.00 | - | 0.95±0.12 | 0.00±0.00 | 0.00±0.00 | - | 0.00±0.00 |
| *GRH6-NIL* | 12.84±3.14 | 0.00±0.00 | 0.00±0.00 | - | 1.63±0.36 | 0.00±0.00 | 0.00±0.00 | - | 0.00±0.00 |
| *GRH2/GRH4-PYL* | 16.67±10.72 | 4.65±2.06 | 2.40±2.40 | 0.58 | 8.24±5.18 | 1.40±1.40 | 104.00±104.00 | 0.60 | 1.39±1.39 |
| *GRH2/GRH5-PYL* | 10.57±4.71 | 1.75±1.06 | 5.20±2.78 | 0.49±0.05 | 10.50±4.67 | 2.00±0.95 | 183.80±121.73 | 0.20±0.04 | 0.85±0.69 |
| *GRH2/GRH6-PYL* | 4.37±2.43 | 1.41±1.09 | 2.60±1.94 | 0.47±0.13 | 7.01±3.46 | 1.40±1.17 | 126.20±119.81 | 0.15±0.15 | 0.80±0.80 |
| *GRH4/GRH5-PYL* | 18.54±12.02 | 0.00±0.00 | 0.00±0.00 | - | 1.28±0.19 | 0.00±0.00 | 0.00±0.00 | - | 0.00±0.00 |
| *GRH4/GRH6-PYL* | 5.70±2.08 | 0.07±0.07 | 0.00±0.00 | - | 1.59±0.46 | 0.00±0.00 | 0.00±0.00 | - | 0.00±0.00 |
| *GRH2/GRH4/GRH6-PYL* | 1.78±1.16 | 5.25±1.50 | 9.40±1.47 | 0.68±0.04 | 23.44±3.20 | 6.00±0.95 | 485.60±96.43 | 0.50±0.12 | 5.85±1.79 |
| 150 Kg N ha^-1^ | B |  | B |  | B | C |  |  |  |
| T65 | 37.17±9.71 | 0.00±0.00 | 0.00±0.00 | - | 1.72±0.28 | 0.00±0.00 | 0.00±0.00 | - | 0.00±0.00 |
| *GRH1-NIL* | 17.46±9.14 | 0.00±0.00 | 0.00±0.00 | - | 1.38±0.20 | 0.00±0.00 | 0.00±0.00 | - | 0.00±0.00 |
| *GRH2-NIL* | 15.95±5.53 | 0.00±0.00 | 0.00±0.00 | - | 1.26±0.21 | 0.00±0.00 | 0.00±0.00 | - | 0.00±0.00 |
| *GRH4-NIL* | 30.23±12.86 | 0.00±0.00 | 0.00±0.00 | - | 0.92±0.22 | 0.00±0.00 | 0.00±0.00 | - | 0.00±0.00 |
| *GRH5-NIL* | 21.51±8.02 | 0.00±0.00 | 0.00±0.00 | - | 0.73±0.19 | 0.00±0.00 | 0.00±0.00 | - | 0.00±0.00 |
| *GRH6-NIL* | 19.09±2.39 | 0.15±0.15 | 0.00±0.00 | - | 2.49±0.82 | 0.00±0.00 | 0.00±0.00 | - | 0.00±0.00 |
| *GRH2/GRH4-PYL* | 6.84±1.19 | 3.19±1.43 | 5.80±1.20 | 0.75±0.07 | 14.70±2.96 | 3.60±1.21 | 257.00±110.12 | - | 2.23±1.08 |
| *GRH2/GRH5-PYL* | 4.54±1.07 | 1.56±0.49 | 6.00±2.59 | 0.65±0.07 | 17.42±4.00 | 3.80±1.59 | 259.40±108.35 | 0.29±0.15 | 1.50±0.98 |
| *GRH2/GRH6-PYL* | 17.13±4.10 | 0.63±0.49 | 1.40±1.40 | 0.14 | 5.84±3.50 | 0.00±0.00 | 0.00±0.00 | - | 0.00±0.00 |
| *GRH4/GRH5-PYL* | 27.93±7.62 | 0.00±0.00 | 0.00±0.00 | - | 1.42±0.20 | 0.00±0.00 | 0.00±0.00 | - | 0.00±0.00 |
| *GRH4/GRH6-PYL* | 9.44±1.03 | 0.20±0.12 | 0.00±0.00 | - | 2.48±0.62 | 0.00±0.00 | 0.00±0.00 | - | 0.00±0.00 |
| *GRH2/GRH4/GRH6-PYL* | 3.75±1.46 | 5.07±2.22 | 9.40±2.25 | 0.83±0.08 | 24.45±3.39 | 6.20±1.02 | 430.20±39.40 | 0.51±0.14 | 4.30±1.32 |
| F_11,284_-Line (L)^b^ | 3.925*** | 1.018ns | 10.242*** |  | 10.150*** | 10.532*** | 6.279*** |  | 4.211*** |
| F_2,284_-Nitrogen (N)^b^ | 5.136** | 0.564ns | 14.478*** |  | 17.015*** | 27.760*** | 2.760ns |  | 0.589ns |
| F_1,284_-Infestation (I)^b^ |  | 381.034*** | 1082.063*** |  | 1275.97*** | 1415.023*** | 805.176*** |  | 282.334*** |
| F_22,284_-L×N^b^ | 1.254ns | 1.071ns | 1.235ns |  | 1.364ns | 1.487ns | 1.449ns |  | 0.981ns |
| F_11,284_-L×I^b^ |  | 4.460*** | 9.757*** |  | 9.836*** | 18.530*** | 10.976*** |  | 5.840*** |
| F_2,284_-N×I^b^ |  | 0.303ns | 10.826*** |  | 10.729*** | 20.991*** | 3.019* |  | 1.489ns |
| F_22,284_-L×N×I^b^ |  | 1.080ns | 0.708ns |  | 1.253ns | 1.418ns | 1.131ns |  | 0.917ns |

a: Numbers are means ± SEM (N = 5); Upper case letters indicate homogenous fertilizer groups, lower case letters indicate homogenous line groups based on Tukey tests (P ≤ 0.05). Note that data presented in Table S3A were included in the analysis.

b: *** = P ≤ 0.001, ** = P ≤ 0.01, * = P ≤ 0.05, ns = P ≥ 0.05. Results indicate that plants grown in pots responded to nitrogen levels by increasing tiller numbers, shoot biomass and panicle weight (controls also had higher numbers of grain). GLH responded to nitrogen levels by gaining higher biomass per weight of shoot (biomass density) on high nitrogen plants. There were few consistent trends in plant weight related to lines under control, non-infested conditions. However, across the experiment *GRH2/GRH4/GRH6*-PYL had higher tiller numbers, greater shoot biomass, more panicles and higher grain production (number) compared to T65. However, in the greenhouse, only *GRH2/GRH5*-PYL had higher grain weights. These trends were mainly due to the lower densities of GLH that developed on the PYLs in the experiment and the strong effects of GLH in reducing plant productivity during the experiment. Significant line × infestation interactions were due to the effects of resistance in maintaining plant production and nitrogen × infestation interactions were due to the similar plant biomass and yields of infested plants irrespective of nitrogen levels, but strong responses to nitrogen by non-infested plants.

***Protocol for greenhouse experiment***

Seed of each of the lines were sterilized and germinated as described above (section 2.4). Germinated seed was transplanted individually to 360 size-6 pots (15×15cm: H×D). The pots were filled with paddy soil collected from the IRRI Experimental Station. Pots were divided into three groups of 120. Two of the groups received basal applications of ammonium sulphate. The plants were tended for 15 days before receiving a second application of nitrogen. The final fertilizer applications simulated field application rates equivalent to 60 or 150 Kg N ha^-1^. A third group of 72 pots received no fertilizer applications. After fertilizer treatments, all plants were enclosed in acetate insect cages (150 × 10cm: H × R). Seventy two pots, two each with one of the 12 lines under one of three nitrogen regimes, were interspersed on a greenhouse bench (experimental block). Blocks were replicated five times.

The plants were allowed to develop for a further 5 days and at 20 DAS (early tillering plants), four gravid female GLH were introduced to half the cages, with corresponding treatments (lines and nitrogen levels) as control, non-infested plants. Pots were monitored until they began to yellow prior to hopperburn, or until they were ready for harvest (> 85% of grain matured), at which time they were sampled. At sampling, GLH were collected from the plants using a vacuum sampler (Hausherr’s Machine Works, USA). The number of tillers were counted (distinguishing productive and non-productive tillers) and the plants destructively harvested. Plant parts were separated as roots, shoots and panicles into paper bags. The plants and GLH were dried in a forced draught oven at 60°C for >1 week and weighed. Grain was removed from the panicles and separated into filled and unfilled grain before counting and final weighing.

For the data analyses, GLH pressure was expressed as the biomass of leafhoppers per unit weight of plant. Data from the experiment was analysed using univariate GLMs with nitrogen regime, line, infestation/control and their interactions as main factors. The effects of ‘block’ (greenhouse bench) were removed. Data residuals were plotted after each analysis and verified as normal and homogeneous.

| **Table S4A**: Results from screen house experiment with eight lines under three nitrogenous fertilizer regimes (equivalent to 0, 60 and 150 Kg N ha^-1^). Plants were not exposed to insect herbivores^a^   \| Line \| Root dry weight (g)^c^ \| Number of tillers \| Proportion of tiller productive^d^ \| Shoot dry weight (g) \| Number of panicles \| Number of grain \| Proportion of filled grain^d^ \| 100 grain weight \| Weight of filled grain (g)^c^ \| \| --- \| --- \| --- \| --- \| --- \| --- \| --- \| --- \| --- \| --- \| \| 0 Kg N ha^-1^ \| A \| A \|  \| A \| A \| A \|  \|  \| A \| \| T65 \| 2.73±0.68 \| 8.25±1.68 \| 0.79±0.02 \| 17.24±3.71 \| 6.25±1.24 \| 533.50±129.47 \| 0.70±0.06 \| 2.48±0.05b \| 9.22±2.14 \| \| *GRH2-NIL* \| 4.72±0.86 \| 8.75±1.28 \| 0.89±0.04 \| 23.61±2.66 \| 5.75±0.37 \| 607.00±71.66 \| 0.61±0.09 \| 2.43±0.15b \| 9.07±2.10 \| \| *GRH4-NIL* \| 4.74±0.56 \| 11.60±1.33 \| 0.85±0.05 \| 27.54±3.37 \| 8.80±1.02 \| 848.80±157.45 \| 0.63±0.10 \| 2.18±0.07ab \| 11.60±2.86 \| \| *GRH5-NIL* \| 4.54±1.11 \| 11.20±1.53 \| 0.86±0.07 \| 22.28±3.91 \| 8.80±2.01 \| 941.00±241.76 \| 0.47±0.04 \| 2.25±0.06a \| 9.87±2.90 \| \| *GRH2/GRH4-PYL* \| 3.89±0.32 \| 7.20±1.02 \| 0.86±0.06 \| 20.11±2.18 \| 5.20±0.49 \| 687.00±79.33 \| 0.57±0.12 \| 2.08±0.06ab \| 8.36±2.58 \| \| *GRH2/GRH5-PYL* \| 2.70±0.50 \| 9.60±1.21 \| 0.69±0.07 \| 16.17±2.09 \| 5.80±0.80 \| 580.00±118.53 \| 0.47±0.13 \| 1.96±0.12a \| 5.75±2.45 \| \| *GRH2/GRH6-PYL* \| 3.85±0.36 \| 12.00±1.90 \| 0.83±0.07 \| 22.20±2.63 \| 8.20±0.37 \| 908.20±28.87 \| 0.48±0.11 \| 2.07±0.09a \| 9.38±2.39 \| \| *GRH2/GRH4/GRH6-PYL* \| 2.50±0.40 \| 10.20±0.86 \| 0.71±0.11 \| 15.40±1.12 \| 6.40±0.60 \| 366.80±28.12 \| 0.52±0.10 \| 2.24±0.03ab \| 4.26±0.86 \| \| 60 Kg N ha^-1^ \| B \| B \|  \| B \| B \| B \|  \|  \| B \| \| T65 \| 7.08±1.47 \| 17.50±3.28 \| 0.70±0.10 \| 36.95±3.67 \| 10.75±1.48 \| 1073.25±156.01 \| 0.54±0.05 \| 2.35±0.04 \| 13.40±1.56 \| \| *GRH2-NIL* \| 7.27±2.94 \| 11.60±3.14 \| 0.78±0.12 \| 28.41±8.17 \| 7.80±2.08 \| 798.40±253.42 \| 0.60±0.09 \| 2.18±0.15 \| 11.22±5.10 \| \| *GRH4-NIL* \| 8.89±2.31 \| 15.80±2.48 \| 0.81±0.08 \| 31.80±3.01 \| 11.00±1.67 \| 854.00±129.66 \| 0.61±0.08 \| 2.25±0.06 \| 11.77±1.56 \| \| *GRH5-NIL* \| 6.22±1.74 \| 18.00±2.95 \| 0.77±0.10 \| 35.08±2.42 \| 11.80±2.15 \| 1327.40±239.04 \| 0.52±0.06 \| 2.09±0.04 \| 14.67±2.92 \| \| *GRH2/GRH4-PYL* \| 9.24±0.87 \| 15.80±3.43 \| 0.78±0.04 \| 38.14±4.76 \| 10.40±2.77 \| 1022.00±206.33 \| 0.62±0.09 \| 2.42±0.09 \| 15.33±2.01 \| \| *GRH2/GRH5-PYL* \| 4.87±0.48 \| 18.20±3.14 \| 0.67±0.12 \| 29.06±2.49 \| 11.80±1.39 \| 1213.00±87.97 \| 0.57±0.05 \| 1.99±0.06 \| 13.72±1.07 \| \| *GRH2/GRH6-PYL* \| 7.82±1.75 \| 15.80±2.58 \| 0.70±0.05 \| 32.98±2.83 \| 10.60±1.47 \| 1264.20±75.53 \| 0.30±0.11 \| 2.04±0.06 \| 7.83±2.82 \| \| *GRH2/GRH4/GRH6-PYL* \| 5.06±0.56 \| 16.80±2.62 \| 0.73±0.10 \| 32.48±3.17 \| 11.60±1.54 \| 884.80±157.99 \| 0.70±0.04 \| 2.35±0.18 \| 14.29±2.06 \| \| 150 Kg N ha^-1^ \| B \| B \|  \| C \| C \| C \|  \|  \| C \| \| T65 \| 6.94±0.73 \| 21.50±3.37 \| 0.72±0.07 \| 49.49±6.61 \| 15.00±1.84 \| 1499.75±194.26 \| 0.60±0.08 \| 2.48±0.04 \| 22.26±0.98 \| \| *GRH2-NIL* \| 8.52±1.89 \| 16.80±2.20 \| 0.75±0.06 \| 40.23±3.03 \| 11.60±0.81 \| 1253.20±126.61 \| 0.68±0.07 \| 2.45±0.07 \| 20.82±3.68 \| \| *GRH4-NIL* \| 6.77±1.99 \| 20.20±1.50 \| 0.70±0.09 \| 32.59±6.41 \| 12.60.2.09 \| 1082.60±179.20 \| 0.73±0.06 \| 2.27±0.14 \| 17.97±4.39 \| \| *GRH5-NIL* \| 6.71±1.00 \| 20.80±4.09 \| 0.86±0.08 \| 41.62±1.38 \| 16.40±1.21 \| 1831.80±177.02 \| 0.63±0.02 \| 1.99±0.06 \| 23.15±2.67 \| \| *GRH2/GRH4-PYL* \| 9.56±1.21 \| 15.40±1.36 \| 0.73±0.11 \| 35.27±3.79 \| 9.60±1.21 \| 1171.40±186.45 \| 0.71±0.05 \| 2.27±0.04 \| 18.90±3.60 \| \| *GRH2/GRH5-PYL* \| 7.24±1.03 \| 17.20±2.85 \| 0.78±0.07 \| 40.50±4.38 \| 12.20±2.22 \| 1227.80±216.54 \| 0.50±0.10 \| 2.14±0.06 \| 13.37±3.80 \| \| *GRH2/GRH6-PYL* \| 9.23±2.11 \| 21.00±2.30 \| 0.70±0.04 \| 41.63±3.61 \| 13.40±1.21 \| 1379.80±218.68 \| 0.70±0.04 \| 2.02±0.04 \| 19.51±4.07 \| \| *GRH2/GRH4/GRH6-PYL* \| 8.08±2.69 \| 17.00±3.18 \| 0.74±0.05 \| 35.72±3.96 \| 10.80±1.91 \| 843.20±141.56 \| 0.54±0.07 \| 2.10±0.04 \| 9.51±1.53 \| \| F_2,119_-Nitrogen (N)^b^ \| 8.563** \| 9.740** \| 0.218ns \| 16.767*** \| 10.156** \| 9.351** \| 1.169ns \| 0.001ns \| 8.404** \| \| F_7,119_-Line (L)^b^ \| 0.554ns \| 0.435ns \| 0.384ns \| 0.325ns \| 0.771ns \| 1.519ns \| 0.733ns \| 2.811* \| 0.618ns \| \| F_14,119_-N×L^b^ \| 0.249ns \| 0.160ns \| 0.257ns \| 0.468ns \| 0.251ns \| 0.299ns \| 0.469ns \| 0.726ns \| 0.411ns \| |  |  |  |  |  |  |  |  |  |
| --- | --- | --- | --- | --- | --- | --- | --- | --- | --- | --- | --- | --- | --- | --- | --- | --- | --- | --- | --- | --- | --- | --- | --- | --- | --- | --- | --- | --- | --- | --- | --- | --- | --- | --- | --- | --- | --- | --- | --- | --- | --- | --- | --- | --- | --- | --- | --- | --- | --- | --- | --- | --- | --- | --- | --- | --- | --- | --- | --- | --- | --- | --- | --- | --- | --- | --- | --- | --- | --- | --- | --- | --- | --- | --- | --- | --- | --- | --- | --- | --- | --- | --- | --- | --- | --- | --- | --- | --- | --- | --- | --- | --- | --- | --- | --- | --- | --- | --- | --- | --- | --- | --- | --- | --- | --- | --- | --- | --- | --- | --- | --- | --- | --- | --- | --- | --- | --- | --- | --- | --- | --- | --- | --- | --- | --- | --- | --- | --- | --- | --- | --- | --- | --- | --- | --- | --- | --- | --- | --- | --- | --- | --- | --- | --- | --- | --- | --- | --- | --- | --- | --- | --- | --- | --- | --- | --- | --- | --- | --- | --- | --- | --- | --- | --- | --- | --- | --- | --- | --- | --- | --- | --- | --- | --- | --- | --- | --- | --- | --- | --- | --- | --- | --- | --- | --- | --- | --- | --- | --- | --- | --- | --- | --- | --- | --- | --- | --- | --- | --- | --- | --- | --- | --- | --- | --- | --- | --- | --- | --- | --- | --- | --- | --- | --- | --- | --- | --- | --- | --- | --- | --- | --- | --- | --- | --- | --- | --- | --- | --- | --- | --- | --- | --- | --- | --- | --- | --- | --- | --- | --- | --- | --- | --- | --- | --- | --- | --- | --- | --- | --- | --- | --- | --- | --- | --- | --- | --- | --- | --- | --- | --- | --- | --- | --- | --- | --- | --- | --- | --- | --- | --- | --- | --- | --- | --- | --- | --- | --- | --- | --- | --- | --- | --- | --- | --- | --- | --- | --- | --- | --- | --- | --- | --- | --- | --- | --- | --- | --- | --- | --- | --- | --- | --- | --- | --- | --- | --- | --- | --- | --- | --- | --- | --- | --- | --- | --- | --- | --- | --- |

a: Numbers are means ± SEM (N = 5); Upper case letters indicate homogenous fertilizer groups, lower case letters indicate homogenous line groups based on Tukey tests (P ≤ 0.05).

b: *** = P ≤ 0.001, ** = P ≤ 0.01, * = P ≤ 0.05, ns = P ≥ 0.05.

c: Data log (x+1) transformed before analysis

d: Data arcsine transformed before analysis

**Table S4B:** Results from screen house experiment with eight lines under three nitrogenous fertilizer regimes (equivalent to 0, 60 and 150 Kg N ha^-1^). Plants were infested by four gravid female *Nephotettix virescens* (GLH) at 20 days after sowing^a^.

| Line | GLH biomass density  (mg/g plant)^c^ | Root dry weight (g)^d^ | Number of tillers | Proportion of tillers productive^e^ | Shoot dry weight (g) | Number of panicles | Number of grain | Proportion of filled grain^e^ | 100 grain weight | Weight of filled grain (g)^d^ |
| --- | --- | --- | --- | --- | --- | --- | --- | --- | --- | --- |
| 0 Kg N ha^-1^ |  | A | A |  | A | A | A |  |  | A |
| T65 | 20.06±4.25d | 0.32±0.14a | 0.00±0.00ab | - | 1.64±0.55a | 0.00±0.00ab | 0.00±0.00abc | - | 0.00±0.00b | 0.00±0.00a |
| *GRH2-NIL* | 23.58±8.10cd | 0.88±0.75ab | 1.80±1.80a | 0.67 | 5.32±3.18a | 1.00±1.00a | 141.40±141.40a | 0.64 | 0.48±0.48b | 2.15±2.15a |
| *GRH4-NIL* | 21.93±7.29cd | 0.50±0.04bcd | 0.00±0.00ab | - | 1.22±0.29a | 0.00±0.00abc | 0.00±0.00ab | - | 0.00±0.00ab | 0.00±0.00a |
| *GRH5-NIL* | 7.65±2.79bc | 0.32±0.17ab | 0.00±0.00ab | - | 1.90±1.33ab | 0.00±0.00abc | 0.00±0.00bcd | - | 0.00±0.00ab | 0.00±0.00ab |
| *GRH2/GRH4-PYL* | 6.85±4.76ab | 2.04±0.38d | 3.60±1.86bc | 0.89 | 9.81±4.98bc | 1.80±0.97bc | 164.00±95.38d | 0.27 | 1.18±0.49ab | 0.87±0.50bc |
| *GRH2/GRH5-PYL* | 0.73±0.61a | 3.66±0.96cd | 9.40±1.96cd | 0.64±0.05 | 22.39±4.54bc | 5.40±1.44cd | 469.80±123.03cd | 0.47±0.09 | 1.98±0.05a | 4.52±1.49bc |
| *GRH2/GRH6-PYL* | 4.83±1.90bc | 1.15±0.38bc | 4.40±1.21abc | 0.82±0.07 | 8.53±2.27abc | 3.40±0.68bc | 236.60±113.26abcd | 0.27 | 1.60±0.41a | 1.31±0.61ab |
| *GRH2/GRH4/GRH6-PYL* | 0.40±0.29a | 3.02±0.49d | 10.20±0.80d | 0.80±0.07 | 19.52±2.68c | 8.20±0.86d | 678.80±98.80cd | 0.65±0.07 | 2.29±0.04ab | 10.14±2.18c |
| 60 Kg N ha^-1^ |  | B | B |  | B | B | B |  |  | B |
| T65 | 28.16±5.60 | 0.08±0.08 | 0.00±0.00 | - | 3.67±0.26 | 0.00±0.00 | 0.00±0.00 | - | 0.00±0.00 | 0.00±0.00 |
| *GRH2-NIL* | 35.89±25.15 | 1.35±0.28 | 0.00±0.00 | - | 5.68±2.03 | 0.00±0.00 | 0.00±0.00 | - | 0.00±0.00 | 0.00±0.00 |
| *GRH4-NIL* | 51.76±19.04 | 9.08±0.08 | 2.60±2.60 | 0.92 | 11.77±9.75 | 2.40±2.40 | 233.00±233.00 | 0.82 | 0.44±0.44 | 4.22±4.22 |
| *GRH5-NIL* | 15.15±6.74 | 1.25±0.12 | 2.00±1.22 | 0.70 | 7.26±1.84 | 1.40±0.98 | 88.80±54.39 | 0.58 | 0.85±0.53 | 1.13±0.77 |
| *GRH2/GRH4-PYL* | 1.35±1.25 | 5.71±1.59 | 11.40±2.98 | 0.82±0.06 | 27.20±4.32 | 8.00±1.90 | 766.00±133.15 | 0.36±0.11 | 2.20±0.08 | 5.88±1.55 |
| *GRH2/GRH5-PYL* | 0.53±0.17 | 4.82±2.02 | 11.00±1.14 | 0.73±0.09 | 25.15±3.91 | 7.20±0.37 | 625.20±50.30 | 0.47±0.13 | 2.01±0.06 | 6.18±2.22 |
| *GRH2/GRH6-PYL* | 10.14±4.12 | 0.78±0.16 | 3.40±1.40 | 0.88 | 6.47±2.20 | 2.80±1.24 | 127.40±53.98 | 0.42 | 1.19±0.49 | 1.07±0.50 |
| *GRH2/GRH4/GRH6-PYL* | 0.50±0.30 | 3.65±0.50 | 13.80±1.91 | 0.77±0.06 | 25.78±2.53 | 11.00±1.30 | 779.60±78.07 | 0.65±0.04 | 2.33±0.10 | 14.10±1.70 |
| 150 Kg N ha^-1^ |  | B | C |  | B | B | C |  |  | B |
| T65 | 28.71±11.92 | 0.00±0.00 | 0.00±0.00 | - | 2.99±0.65 | 0.00±0.00 | 0.00±0.00 | - | 0.00±0.00 | 0.00±0.00 |
| *GRH2-NIL* | 35.99±18.12 | 0.68±0.31 | 0.00±0.00 | - | 5.11±2.23 | 0.00±0.00 | 0.00±0.00 | - | 0.00±0.00 | 0.00±0.00 |
| *GRH4-NIL* | 12.02±5.91 | 0.91±0.25 | 0.00±0.00 | - | 5.86±2.50 | 0.00±0.00 | 0.00±0.00 | - | 0.00±0.00 | 0.00±0.00 |
| *GRH5-NIL* | 3.84±1.25 | 1.92±1.20 | 4.60±4.60 | 0.83 | 15.80±7.64 | 2.40±2.40 | 220.00±220.00 | 0.36 | 0.42±0.42 | 1.66±1.66 |
| *GRH2/GRH4-PYL* | 7.92±4.20 | 5.87±1.46 | 10.80±1.98 | 0.98±0.01 | 29.01±5.69 | 9.20±1.07 | 838.40±218.83 | 0.48±0.13 | 1.92±0.19 | 8.84±4.87 |
| *GRH2/GRH5-PYL* | 0.42±0.22 | 6.19±2.20 | 17.40±4.76 | 0.62±0.08 | 30.06±4.73 | 6.40±0.93 | 494.80±102.21 | 0.53±0.08 | 1.96±0.07 | 5.22±1.77 |
| *GRH2/GRH6-PYL* | 5.01±1.43 | 1.87±0.32 | 4.80±1.98 | 1.00 | 18.65±2.58 | 4.80±1.98 | 285.20±126.78 | 0.39 | 1.22±0.50 | 2.28±1.01 |
| *GRH2/GRH4/GRH6-PYL* | 1.43±0.52 | 9.88±3.57 | 22.00±3.59 | 0.65±0.08 | 37.51±6.63 | 12.40±1.78 | 1001.40±243.25 | 0.55±0.11 | 2.20±0.03 | 12.02±3.74 |
| F_2,155_-Nitrogen (N)^b^ | 0.109ns | 19.914*** | 17.703*** | 0.089ns | 28.569*** | 18.143*** | 15.478*** | 1.215ns | 0.550ns | 7.928** |
| F_7,155_-Infested (I)^b^ |  | 134.410*** | 104.556*** | 0.266ns | 126.314*** | 132.735*** | 150.884*** | 2.492ns | 206.001*** | 209.889*** |
| F_2,155_-N×I^b^ |  | 1.222ns | 3.080ns | 0.632ns | 3.528ns | 4.012* | 5.258** | 1.011ns | 0.571ns | 2.785ns |
| F_7,155_-Line (L)^b^ | 6.271** | 8.067*** | 5.040*** | 0.804ns | 3.601*** | 5.008*** | 2.644** | 1.301n | 13.344*** | 5.348*** |
| F_14,155_-N×L^b^ | 0.277ns | 1.789ns | 0.402ns | 0.537ns | 0.769ns | 0.564ns | 0.528ns | 0.832ns | 0.761ns | 0.890ns |
| F_7,155_-V×I^b^ |  | 8.625*** | 6.108*** | 1.778ns | 7.139*** | 7.782*** | 7.349*** | 0.539ns | 18.225*** | 10.389*** |
| F_14,155_-N×L×I^b^ |  | 1.445ns | 0.635ns | 0.264ns | 0.728ns | 0.551ns | 0.660ns | 0.246ns | 0.412ns | 0.441ns |

a: Numbers are means ± SEM (N = 5); Upper case letters indicate homogenous fertilizer groups, lower case letters indicate homogenous line groups based on Tukey tests (P ≤ 0.05). Note that data presented in Table S4A were included in the analysis (split-split plot design).

b: *** = P ≤ 0.001, ** = P ≤ 0.01, * = P ≤ 0.05, ns = P ≥ 0.05.

c: Data ranked before analysis

d: Data log (x+1) transformed before analysis

e: Data arcsine transformed before analysis

**Table S5:** Growth and yield of 15 lines in field plots under two nitrogenous fertilizer regimes (equivalent to 0 and 60 Kg N ha^-1^).

| Variety | Number of tillers | Proportion of tillers productive | Shoot dry weight (g) | Number of panicles | Number of grain | Proportion of grain filled | 100 grain weight | Weight of filled grain (g) |
| --- | --- | --- | --- | --- | --- | --- | --- | --- |
| 0 Kg N ha^-1^ |  |  |  |  |  |  |  |  |
| *T65* | 16.00±1.88 | 83.89±6.77 | 24.71±1.68 | 12.50±1.52 | 909.83±92.98 | 77.15±7.18 | 2.32±0.07 | 17.39±3.50 |
| *GRH1-NIL* | 10.17±1.49 | 93.14±6.86 | 20.91±2.57 | 8.33±0.80 | 966.56±149.69 | 83.73±4.24 | 2.36±0.16 | 14.86±2.57 |
| *GRH2-NIL* | 11.67±0.88 | 97.00±1.94 | 22.96±3.54 | 10.83±1.08 | 708.17±69.65 | 81.21±6.43 | 2.26±0.10 | 17.73±3.09 |
| *GRH4-NIL* | 12.20±2.01 | 93.48±3.47 | 18.58±1.85 | 10.00±1.24 | 778.33±147.75 | 80.06±3.62 | 2.19±0.02 | 13.18±1.41 |
| *GRH5-NIL* | 11.60±1.89 | 90.50±4.19 | 19.37±2.48 | 9.00±1.13 | 837.50±113.72 | 75.61±2.71 | 2.08±0.10 | 12.13±1.77 |
| *GRH6-NIL* | 13.17±1.62 | 93.65±4.43 | 16.87±2.55 | 11.33±0.92 | 880.00±62.54 | 67.66±4.22 | 2.05±0.10 | 12.96±2.72 |
| *qGRH4-NIL* | 12.67±1.58 | 100.00±0.00 | 18.79±1.41 | 12.00±1.34 | 883.65±62.36 | 77.88±3.58 | 2.14±0.06 | 15.07±1.66 |
| *GRH2/GRH4-PYL* | 12.50±0.43 | 81.58±7.27 | 24.40±2.58 | 8.67±0.80 | 924.17±97.34 | 77.93±4.48 | 2.14±0.13 | 16.67±3.40 |
| *GRH2/GRH5-PYL* | 15.00±1.39 | 71.42±4.34 | 17.20±0.90 | 9.00±0.77 | 943.00±118.08 | 77.88±5.68 | 2.09±0.10 | 9.73±1.48 |
| *GRH2/GRH6-PYL* | 12.83±1.38 | 91.45±5.41 | 18.11±2.76 | 10.83±1.11 | 579.83±44.01 | 68.09±11.16 | 5.31±3.06 | 13.86±2.64 |
| *GRH4,/GRH5-PYL* | 15.67±3.20 | 96.22±3.03 | 21.74±2.98 | 12.67±1.89 | 853.20±108.41 | 80.21±6.25 | 2.06±0.09 | 14.29±1.95 |
| *GRH4,/GRH6-PYL* | 14.00±2.22 | 85.07±6.00 | 21.53±3.54 | 11.00±1.41 | 863.83±104.18 | 81.51±5.40 | 2.33±0.09 | 16.22±2.95 |
| *GRH5,/GRH4-PYL* | 18.25±2.40 | 90.79±4.47 | 22.55±1.48 | 14.75±1.62 | 774.8±94.34 | 86.45±2.06 | 2.18±0.03 | 16.53±1.09 |
| *GRH2/GRH4/GRH6-PYL* | 18.80±2.52 | 83.26±5.23 | 22.51±3.47 | 13.80±1.58 | 771.80±115.35 | 74.63±4.84 | 2.24±0.08 | 14.51±2.40 |
| 60 Kg N ha^-1^ |  |  |  |  |  |  |  |  |
| *T65* | 18.33±2.80 | 96.42±2.50 | 32.65±4.10 | 17.00±2.31 | 1449.33±217.00 | 88.65±1.22 | 2.40±0.05 | 28.42±2.69 |
| *GRH1-NIL* | 14.33±1.17 | 90.76±4.15 | 28.13±2.14 | 12.83±0.79 | 1346.36±150.90 | 84.39±2.62 | 2.49±0.11 | 21.97±2.36 |
| *GRH2-NIL* | 16.67±1.86 | 93.94±6.06 | 30.23±2.75 | 15.33±1.54 | 1039.83±83.19 | 87.55±1.09 | 2.41±0.05 | 28.34±2.33 |
| *GRH4-NIL* | 21.50±5.58 | 84.11±10.24 | 28.92±3.50 | 14.83±1.92 | 1350.67±83.90 | 86.81±2.79 | 2.23±0.07 | 24.37±1.91 |
| *GRH5-NIL* | 12.67±1.71 | 95.02±3.30 | 23.40±3.15 | 11.83±1.64 | 1308.17±87.83 | 71.75±6.36 | 2.09±0.09 | 17.86±3.48 |
| *GRH6-NIL* | 17.33±2.29 | 95.38±3.13 | 24.63±2.35 | 16.17±1.87 | 1283.84±69.30 | 75.19±2.33 | 2.16±0.10 | 25.12±2.90 |
| *qGRH4-NIL* | 21.17±5.12 | 93.70±6.30 | 32.27±5.60 | 18.17±2.63 | 1546.83±158.65 | 86.05±2.97 | 2.14±0.07 | 26.25±3.76 |
| *GRH2/GRH4-PYL* | 14.67±2.11 | 98.25±1.75 | 29.06±3.69 | 13.50±2.06 | 1350.17±121.14 | 81.63±3.52 | 2.13±0.10 | 25.04±5.26 |
| *GRH2/GRH5-PYL* | 30.17±4.68 | 60.84±7.03 | 29.65±4.86 | 16.50±2.78 | 1420.92±237.56 | 79.00±4.91 | 2.18±0.05 | 22.42±4.82 |
| *GRH2/GRH6-PYL* | 18.50±1.34 | 86.87±4.94 | 24.29±1.35 | 15.83±1.40 | 1237.17±233.28 | 80.45±2.25 | 2.21±0.06 | 24.03±1.86 |
| *GRH4,/GRH5-PYL* | 19.00±2.65 | 96.03±3.97 | 32.58±4.37 | 18.17±2.65 | 1215.33±104.61 | 80.56±3.60 | 2.04±0.06 | 26.27±4.75 |
| *GRH4,/GRH6-PYL* | 19.17±2.21 | 93.34±4.27 | 26.82±3.66 | 17.33±1.36 | 1565.50±238.14 | 84.65±2.46 | 2.37±0.06 | 26.58±2.71 |
| *GRH5,/GRH4-PYL* | 21.17±2.60 | 93.33±4.41 | 26.69±2.80 | 18.83±1.92 | 1117.36±150.71 | 82.85±2.72 | 2.10±0.05 | 22.46±1.96 |
| *GRH2/GRH4/GRH6-PYL* | 24.00±1.91 | 83.56±5.10 | 29.56±2.40 | 19.50±1.57 | 1265.50±94.97 | 83.27±1.63 | 2.36±0.02 | 23.88±2.06 |
| F_1,167_-Nitrogen (N) | 3.469ns | 0.118ns | 5.126ns | 7.392* | 11.168* | 0.632ns | 0.067ns | 9.591* |
| F_13,167_-Line (L) | 0.343ns | 0.632ns | 0.138ns | 0.344ns | 0.167ns | 0.255ns | 0.115ns | 0.143ns |
| F_13,167_-N×L | 0.117ns | 0.135ns | 0.055ns | 0.026ns | 0.052ns | 0.073ns | 0.111ns | 0.036ns |

a: Numbers are means ± SEM (N = 6)

b: * = P ≤ 0.05, ns = P ≥ 0.05 (split plot design)
